# Hybrid Silver–Silica and Organic Biocide Systems in PVC: Enhanced Antiviral Performance against SARS-CoV‑2

**DOI:** 10.1021/acsami.5c12270

**Published:** 2025-09-24

**Authors:** Daniel J. da Silva, Guilherme B. Gramcianinov, Vanessa B. Malaquias, Pamela Z. Jorge, Cecilia Gonsales, Eduardo W. A. Pereira, Leice G. Amurin, Mário Hiroyuki Hirata, Caroline C. Augusto, Bruno L. Batista, Beatriz B. Alves, Luciano A. Bueno, Danilo J. Carastan, Mathilde Champeau

**Affiliations:** † Center for Engineering, Modeling and Applied Social Sciences (CECS), 74362 Federal University of ABC (UFABC), Av. dos Estados, 5001, Santo André, SP CEP 09210-580, Brazil; ‡ Department of Clinical and Toxicological Analysis, Faculty of Pharmaceutical Sciences, University of São Paulo, Av. Professor Lineu Prestes, 580, São Paulo, SP CEP 05508-900, Brazil; § Center for Natural and Human Sciences (CCNH), Federal University of ABC (UFABC), Av. dos Estados, 5001, Santo André, SP CEP 09210-580, Brazil; ∥ BR Goods Indústria e Comércio de Produtos Hospitalares, R. Antônio Barnabé, 1398, Indaiatuba, SP CEP: 13347-340, Brazil

**Keywords:** SARS-CoV-2, poly(vinyl chloride), silver, SiO_2_, hybrid nanoparticles, organic biocides

## Abstract

Poly­(vinyl chloride) (PVC) is widely used in biomedical devices and hospital infrastructure. In light of emerging pathogens such as SARS-CoV-2, there is a growing need for PVC materials with intrinsic antiviral properties. This study presents an effective strategy to develop antiviral PVC nanocomposites by incorporating silver–silica (Ag/SiO_2_) hybrid nanoparticles via melt processing. Two hybrid nanoparticle systems were incorporated: SiO_2_ decorated with silver nanoparticles and a mixture of Ag/SiO_2_ with two organic biocides (triclosan and zinc pyrithione). Comprehensive morphological, optical, thermal, and mechanical characterizations were conducted to assess structure–property relationships, along with antiviral tests against SARS-CoV-2. The addition of Ag/SiO_2_ nanoparticles preserved the PVC thermal stability and impact strength, whereas it increased the stiffness. The particles containing the biocides limited PVC discoloration but affected the mechanical properties due to their plasticizing effect. Whereas a concentration of 2.5 wt % of Ag/SiO_2_ nanoparticles inactivated SARS-CoV-2, only 0.5 wt % of the Ag/SiO_2_/organic biocide system was necessary, showing a combined virucidal response between Ag on the nanocomposite surface and triclosan that is released. Excessive filler content led to performance drops or cytotoxic effects. The combination of inorganic and organic antimicrobial agents enables tailored functionality, demonstrating the potential of these materials for use in medical devices, personal protective equipment, and surfaces requiring antimicrobial protection.

## Graphical abstract

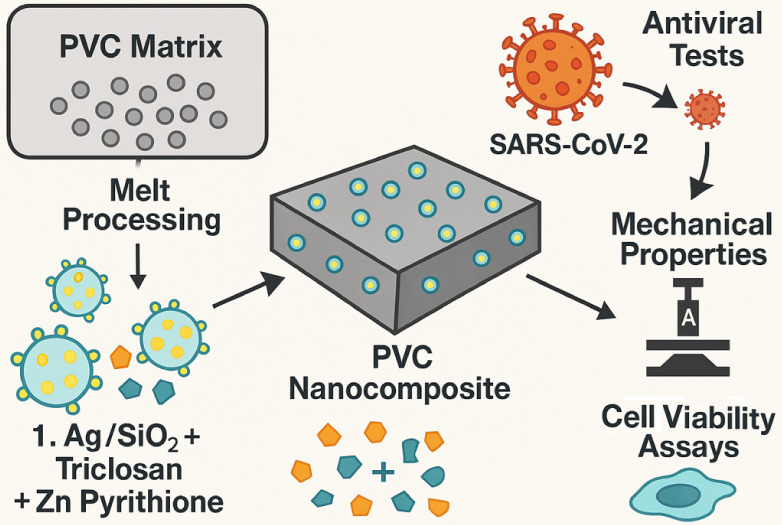

## 1 Introduction

The global pandemic of Coronavirus Disease 2019 (COVID-19), caused by Severe Acute Respiratory Syndrome Coronavirus 2 (SARS-CoV-2) has resulted in significant loss of human lives, leading to the collapse of health systems in developed and developing-countries.^1,2^ In addition to showing the weaknesses of epidemiological control systems, there are several concerns about the secure and adequate protection of the population and health agents against this coronavirus and its more transmissible variants that have emerged in recent years through the use of personal protective equipment (PPE) to prevent a new coronavirus pandemic.^3^ Thus, different efforts from scientific and industrial communities have been carried out to develop new self-disinfecting materials capable of destroying quickly infectious SARS-CoV-2 particles that can be transmitted to human beings by aerosol, tears, secretions, blood, or direct contact with contaminated surfaces.^4,5^

Poly­(vinyl chloride) (PVC) has been widely used in the manufacture of instruments used in examinations, surgeries, and recovery of patients, such as blood bags, endotracheal tubes, saline bags, catheters, and cardiovascular tubes, among others.^6^ It is also used in hospital infrastructure, such as handrails and wall bumpers. PVC is a versatile thermoplastic but is prone to thermomechanical degradation during processing (e.g., extrusion and injection molding), releasing HCl, which can cause irreversible damage to the machinery, shortening its service life. Therefore, stabilizers and processing additives are essential to molding PVC by thermomechanical processes, avoiding the release of acid and preventing an undesirable desiccant degradation, which leads to the drop of mechanical properties and darkening of the PVC due to the formation of conjugated double bonds after zipper dehydrochlorination.^7,8^ However, the addition of processing additives, mainly phthalate ester plasticizers,^9^ makes PVC more susceptible to biofouling, and the intrinsic bactericidal properties of the polymer associated with the HCl generation are lost.^2^

Silver and other metals and oxides (e.g., copper, gold, TiO_2_, Cu_2_O, and CuO) are intrinsically biocide additives and are widely applied to impart antimicrobial properties to different materials,^10,11^ including PVC.^12–16^ In contrast to CuO- and TiO_2_-based nanocomposites, whose antimicrobial activity is typically attributed to Cu^2+^ dissolution and photogenerated reactive oxygen species (ROS), respectively, the antimicrobial and antiviral activity in silver-based nanocomposites is dominated by contact-mediated processes at the material–microbe or material–virus interface. Upon contact, cell membranes and viral envelopes are disrupted, and key surface proteins are inactivated, leading to loss of viability or infectivity. Dissolved ions and ROS may also contribute, but their roles are secondary and system dependent.^10,11,17^ Silver, in particular, can also be incorporated into Ag/oxide hybrid nanoparticles due to its unique ability to modulate the oxide bandgap and suppress electron–hole pair recombination via surface plasmon resonance effects. This interaction significantly enhances the photocatalytic and antimicrobial efficiency of the oxide.^18,19^

Silicon dioxide (SiO_2_), also known as silica, is an abundant oxide on Earth’s crust, being applied by the polymer processing industry as a filler due to its low cost, reducing the final price of the polymer product. For this reason, silica is also used as a support material for more expensive intrinsic antimicrobial agents, such as silver. Moreover, silica is considered safe for human beings. Balagna et al.^20^ showed that silica combined with silver ensures the deactivation of SARS-CoV-2 infectious particles from personal protective masks within 72 h. In particular, highly porous, nanostructured grades of silica particles, such as pyrogenic silica, can provide support for silver nanoparticles, resulting in hybrid nanomaterials with a high surface area that can be combined with polymers to form functional, antimicrobial nanocomposites. Kokate et al.^21^ demonstrated that porous silica support plays a critical role in improving the photocatalytic and bactericidal activities of Ag/SiO_2_/ZnO hybrid nanoparticles by offering a high contact area to this catalytic nanocomposite system.

In addition to metallic and oxide-based systems, biocidal organic compounds have been explored for their complementary antimicrobial mechanisms. Zinc pyrithione (ZPT), a coordination complex of zinc with pyrithione ligands, exhibits strong antimicrobial activity attributed to its ability to act as a Zn^2+^ ionophore, facilitating intracellular zinc accumulation and generating reactive oxygen species (ROS) that disrupt cellular and viral processes.^22,23^ Triclosan (5-chloro-2-(2,4-dichlorophenoxy)­phenol) is another broad-spectrum antimicrobial agent that interferes with lipid synthesis pathways and disrupts the lipid membranes of bacterial and viral pathogens.^24,25^ The incorporation of these biocides into hybrid nanoparticles offers a promising strategy to combine inorganic and organic mechanisms for an enhanced antimicrobial performance.

Although several studies have produced Ag/PVC composites by casting and other methods involving solvents,^26,27^ injection and extrusion molding technologies offer significant advantages for the fabrication of polymeric composites. These include the elimination of toxic solvents, higher dimensional precision, the ability to produce complex geometries, enhanced reproducibility, versatility, and high productivity – factors that are critical engineering parameters in the industrial polymer processing sector. Nonetheless, antimicrobial polymer composites prepared by this route in the molten state generally show only bacteriostatic activity, requiring the use of large amounts of intrinsic antimicrobial agents to improve bactericidal properties. For this reason, silica can be used as inexpensive support for these relatively more expensive agents and thus make it possible to obtain bactericidal polymer compounds at commercially competitive prices.

In this contribution, two hybrid antimicrobial nanoparticle systems (Ag/SiO_2_ and Ag/SiO_2_/ZPT/TCS) were selected for their commercial availability in Brazil and their documented antiviral and antimicrobial properties.^15,28^ The Ag/SiO_2_ system is primarily inorganic and exhibits high thermal stability. In contrast, the Ag/SiO_2_/ZPT/TCS system combines silver, fumed silica, and two organic compounds: zinc pyrithione (ZPT) and triclosan (TCS) (Figure 1). This design enables the assessment of different antimicrobial mechanisms: a predominantly inorganic approach (Ag/SiO_2_) versus a hybrid organic–inorganic strategy (Ag/SiO_2_/ZPT/TCS), both incorporated into the PVC matrix via melt compounding. The resulting samples were characterized in terms of morphology, thermal stability, mechanical properties, and toxicity, as well as their antiviral activity against SARS-CoV-2.

**Figure 1:**
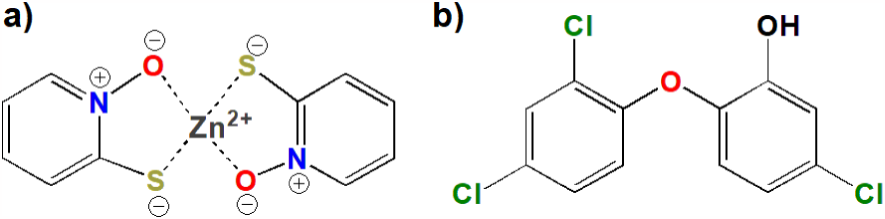
Molecular structure of (a) ZPT and (b) TCS.

## 2 Materials and Methods

### 2.1 Materials

PVC was supplied by Karina Plásticos (Guarulhos, Brazil). Ag/SiO_2_ (Nanox NNXC AB) and Ag/SiO_2_/ZPT/TCS (Nanox NNXC ATZ) powders were purchased from Nanox Tecnologia SA (Brazil). The compounds HNO_3_ (65%), AgNO_3_ (99%), KSCN (>99%), Zn­(NO_3_)_2_·6H_2_O (96–103%), Cu­(NO_3_)_2_·3H_2_O (98–102%), and Fe­(NO_3_)_3_·9H_2_O (≥99.95%) were purchased from Synth (Brazil). All reagents were used as purchased without prior purification. Eagle’s Minimal Essential Medium (EMEM) and 3-[4,5-dimethylthiazol-2-yl]-2,5-diphenyl tetrazolium bromide were purchased from Sigma-Aldrich, a mixture penicillin/streptomycin was bought from Gibco. CO_2_ was purchased from Oxidetoni (Santo André, Brazil).

### 2.2 Methods

#### 2.2.1 Preparation of the PVC/Ag/SiO_2_ and PVC/Ag/SiO_2_/ZPT/TCS Nanocomposites

The PVC nanocomposites were prepared through melt processing in an internal mixer (Model 50EHT 3Z, Brabender GmbH & Co. KG, Germany) at 160 °C and 60 rpm of rotor speed. The temperature, rotor speed and fill factor were optimized after previous experiments, to maximize nanoparticle dispersion and to avoid PVC degradation. First, PVC was plasticized for 2 min before mixing with the antimicrobial powders (internal mixer fill factor = 80%). The PVC compounds were then mixed for 8 min. The nanocomposites were named PVC/*X*(Ag/SiO_2_) and PVC/*X*(Ag/SiO_2_/ZPT/TCS), where *X* corresponds to the concentration (in wt %) of the antimicrobial agent system (Ag/SiO_2_ and Ag/SiO_2_/ZPT/TCS).

Impact test specimens were injection molded at 180 °C using a Micro Injection Molder (Model 12 cm^3^, XPlore Instruments BV, The Netherlands) with an injection pressure of 9 bar. The injection cycle included a plasticizing residence time of 150 s, injection time of 8 s, pressure time of 10 s, and holding time of 15 s. The mold temperature was set to 40 °C. The specimen geometry for impact testing followed the ASTM D256 standard.

The tensile test specimens were prepared in a hydraulic press (model SL 11, Solab Científica, Brazil) using a 1 mm thick mold at 190 °C. The material was preheated for 3 min, followed by compression at 6 tons for 5 min. The films were then wedge-cut into specimen shapes in accordance with ASTM D1708.

### 2.3 Characterization

#### 2.3.1 Ag/SiO_2_ and Ag/SiO_2_/ZPT/TCS Hybrid Nanoparticles

##### 2.3.1.1 Scanning Electron Microscopy (SEM)

SEM micrographs were obtained using a JEOL compact scanning electron microscope (JSM-6010LA) using a secondary electron detector (SEI) and an accelerating voltage of 10 kV. The nanoparticles were directly deposited onto a carbon tape. The particles were sputter-coated with a 5 nm-thick gold layer.

##### 2.3.1.2 Transmission Electron Microscopy (TEM)

Suspensions of the hybrid nanoparticle systems were prepared in isopropanol at a concentration of 0.1 mg/mL and dispersed using an ultrasonic bath (Elma P70H) for 20 min at 30 °C. The resulting dispersions were gently dropped onto 200-mesh copper grids coated with a carbon film and left to dry at room temperature. TEM images of the nanofillers were obtained using a Talos F200X G2 high-resolution transmission electron microscope (HRTEM) operated at an accelerating voltage of 200 kV. Imaging was performed using various detectors, including bright-field (BF), high-angle annular dark-field (HAADF), and energy-dispersive X-ray spectroscopy (EDS) for elemental analysis.

##### 2.3.1.3 Thermogravimetric Analysis (TGA)

Thermal stability was evaluated by thermogravimetric analysis (TGA), using the STA 6000 thermogravimetric analyzer (PerkinElmer) and alumina pans. The samples were heated from 30 to 500 °C at a heating rate of 10 °C min^–1^ under N_2_ flow (gas flow = 20 mL min^–1^). The maximum thermal decomposition temperature (*T*_max_) was determined by the peak in the derivative thermogravimetric (DTG) curve. The thermal decomposition onset temperature (*T*_5%_) was defined as the temperature at 5% mass loss in the TGA curves. The composition analysis was performed according to the ASTM E1131-20 standard.

##### 2.3.1.4 Fourier-Transform Infrared Absorption Spectroscopy (FTIR)

Fourier-transform infrared absorption spectroscopy (FTIR) measurements were performed on a Thermo IS5 Nicolet spectrometer, using an attenuated total reflectance (ATR) accessory (ZnSe crystal). Spectral data acquisition was conducted in the 600–4000 cm^–1^, using 32 scans and a spectral resolution of 2 cm^–1^.

##### 2.3.1.5 Inductively Coupled Plasma Atomic Emission Optical Spectrometry (ICP-OES)

The silver, zinc, and copper content in the powders were quantitatively determined in an ICP-OES Axial View, model 710 Series (Varian). The instrumental conditions are detailed in Table S1. The calibration curve was prepared from AgNO_3_, Cu­(NO_3_)_2_, and Zn­(NO_3_)_2_ aqueous solutions (HNO_3_—3%). Copper was not detected in neither of the powders.

#### 2.3.2 PVC Composites

##### 2.3.2.1 Scanning Electron Microscopy (SEM)

The PVC composites were coated with a 20 nm thick gold layer using a Leica EM ACE 200 sputter coater (Leica Microsystems, Wetzlar, Germany). Micrographs were obtained in a FEI Quanta 250 scanning electron microscope (Thermo Fisher Scientific, Hillsboro, Oregon, USA), using an accelerating voltage of 10 kV, a spot size of 4 nm, and a magnification of 5000×.

##### 2.3.2.2 UV–Vis Diffuse Reflectance Spectroscopy

The diffuse reflectance (R_d_) spectra were measured in a UV–vis spectrophotometer (Model Evolution 220, Thermo Fisher, USA). Spectralon was applied as a white reflection pattern since it is a diffuse reflectance material based on polytetrafluoroethylene (PTFE) with *R*_d_ of 100%. These measurements were made in the 200 to 1000 nm range, using a spectral resolution of 1 nm. The yellowness index (YI) was calculated from the reflectance measurements by eq 1.

1 $$YI=\frac{\left(R+G\right)}{B^{2}}$$

Where *R*, *G*, and *B* are reflectance intensity at 680, 530, and 470 nm, respectively.

The band gap energy (*E*_g_) of the PVC samples was estimated from *R*_d_ data (in %) using Tauc’s plots of the Kubelka–Munk function determined by eq 2, as detailed in the literature.^29^

2 $$F\left(R_{d}\right)=\frac{{\left(100-R_{d}\right)}^{2}}{2R_{d}}$$

##### 2.3.2.3 Mechanical Properties

Uniaxial tensile tests were carried out in a universal testing machine (Instron, Model 3367, USA). The mechanical properties were measured using a 50 kN load cell at a test speed of 1.5 mm min^–1^ according to ASTM D1708 (micro tensile test).

Notched Izod impact tests were performed in an Izod Impact Tester (Shanta Engineering, India) at room temperature (25 °C) using a 2.71 J hammer pendulum, according to ASTM 256D – method A. All mechanical data were determined using at least 5 testing specimens.

##### 2.3.2.4 Thermogravimetric Analysis (TGA)

The thermal stability of the polymeric samples was evaluated by a TGA thermal analyzer (Mettler Toledo, USA) using alumina pans. The samples were heated from 50 to 500 °C at a rate of 10 °C min^–1^ under an N_2_ atmosphere (50 mL min^–1^).

##### 2.3.2.5 Aqueous Release of Organic and Inorganic Species

PVC composite samples (1 cm^2^) were cut under laminar flow with sterile scissors, decontaminated with 70% ethanol, packed in surgical-grade paper, sterilized for 20 min at 121 °C under saturated steam at 110 kPa (autoclave pressure), and subsequently dried in an oven at 51 °C for 4 h. PVC nanocomposite films (thickness ≈ 0.05 mm) were immersed in ultrapure water at 37 °C and aliquots were collected after 30, 60, and 120 min. All extractions and analyses were performed in triplicate (*n* = 3).

The silver and zinc content in the aliquots were quantitatively determined in an inductively coupled plasma mass spectrometer (ICP-MS—Agilent 7900, Hachioji, Japan). The instrumental conditions are detailed in Table S4. The triclosan (TCS) content in the aliquots was qualitatively determined by UV–Vis spectroscopy (Cary 50 diode-array, Varian) using 1 cm quartz cuvettes. Spectra (200–600 nm) were baseline-corrected against matched blanks.

##### 2.3.2.6 Antiviral and Cell Viability Assessment

Surface antiviral tests were performed in triplicate according to ISO 21702:2019 standard, at the Laboratory with Biosafety Level 3. PVC composite samples (5 cm^2^) were cut under laminar flow with sterile scissors, decontaminated with 70% ethanol, packed in surgical-grade paper, sterilized for 20 min at 121 °C under saturated steam at 110 kPa pressure (autoclave), and subsequently dried in an oven at 51 °C for 4 h. The SARS-CoV-2 virus (strain B.1.1.28, GenBank accession number: MW441768.1) was titrated according to the TCID_50_ method at 2.5 × 10^6^ TCID_50_/mL.^30^

Vero E6 cell line (ATCC – CRL1586) was cultured using Eagle’s Minimal Essential Medium (EMEM, Sigma-Aldrich) containing 2–10% fetal bovine serum and 1% penicillin/streptomycin (Gibco) in a 5% CO_2_ incubator at 37 °C. Cells were transferred to 96-well plates at 1 × 10^5^ cells/well and incubated until reaching 80–90% confluence. For sample contamination, 100 μL of virus was added to the center of each sample and spread with a sterile disposable loop. Samples were incubated at room temperature for 30, 60, and 120 min. The virus was recovered with a sterile swab, added to Falcon tubes containing 0.9 mL of EMEM medium, vortexed for 1 min, and 150 μL was plated in triplicate on Vero E6 cells. Plates were incubated at 37 °C in a 5% CO_2_ incubator. After 48 h of incubation, the antiviral activity was evaluated through the cytopathic effect and cell viability assessment using the MTT (3-[4,5-dimethylthiazol-2-yl]-2,5-diphenyl tetrazolium bromide) colorimetric assay. Cell viability was measured at 570 nm using SkanIt Software 2.4.5, Varioskan Flash (Thermo Fisher, USA). Results are expressed in percentage of viral inactivation through cell viability compared to cellular controls. The percentage of viral inactivation considered for virucidal activity is >99.99% (>4-log) according to De Vries (1999).^31^

##### 2.3.2.7 Statistical Analysis

One-way analysis of variance (ANOVA two-way), Dunn’s variance test, and Tukey’s test were applied to statistically evaluate the significant differences between the properties of the samples measured, using Origin 2016 and a 95% confidence level.

## 3 Results and Discussion

### 3.1 Characterization of the Ag/SiO_2_ and Ag/SiO_2_/ZPT/TCS Powders

#### 3.1.1 Scanning Electron Microscopy and EDS

The SEM micrographs of the Ag/SiO_2_ and Ag/SiO_2_/ZPT/TCS powders are shown in Figure 2 and in Figures S1 and S2, as well as their diameter distribution. According to these results, the Ag/SiO_2_ powder consists predominantly of particles with an average diameter of 1.1 ± 0.1 μm, particularly with two populations around 0.35 and 2.5 μm. The particle diameters of the Ag/SiO_2_ powder follow a logistic-type distribution, with a fitting error (R^2^) equal to 0.999. The calculated fit curve and the logistic function used in the fit are shown in Figure 2c, while the fitting parameters are detailed in Table S2. Regarding the Ag/SiO_2_/ZPT/TCS powder, it mainly consists of particles with diameters smaller than 361 nm (Figure 2d,e). The average particle diameter of Ag/SiO_2_/ZPT/TCS powder is 1.9 ± 0.1 μm, according to the diameter distribution fitting curve (also logistic function) shown in Figure 2f (fitting parameters in Table S2).

**Figure 2:**
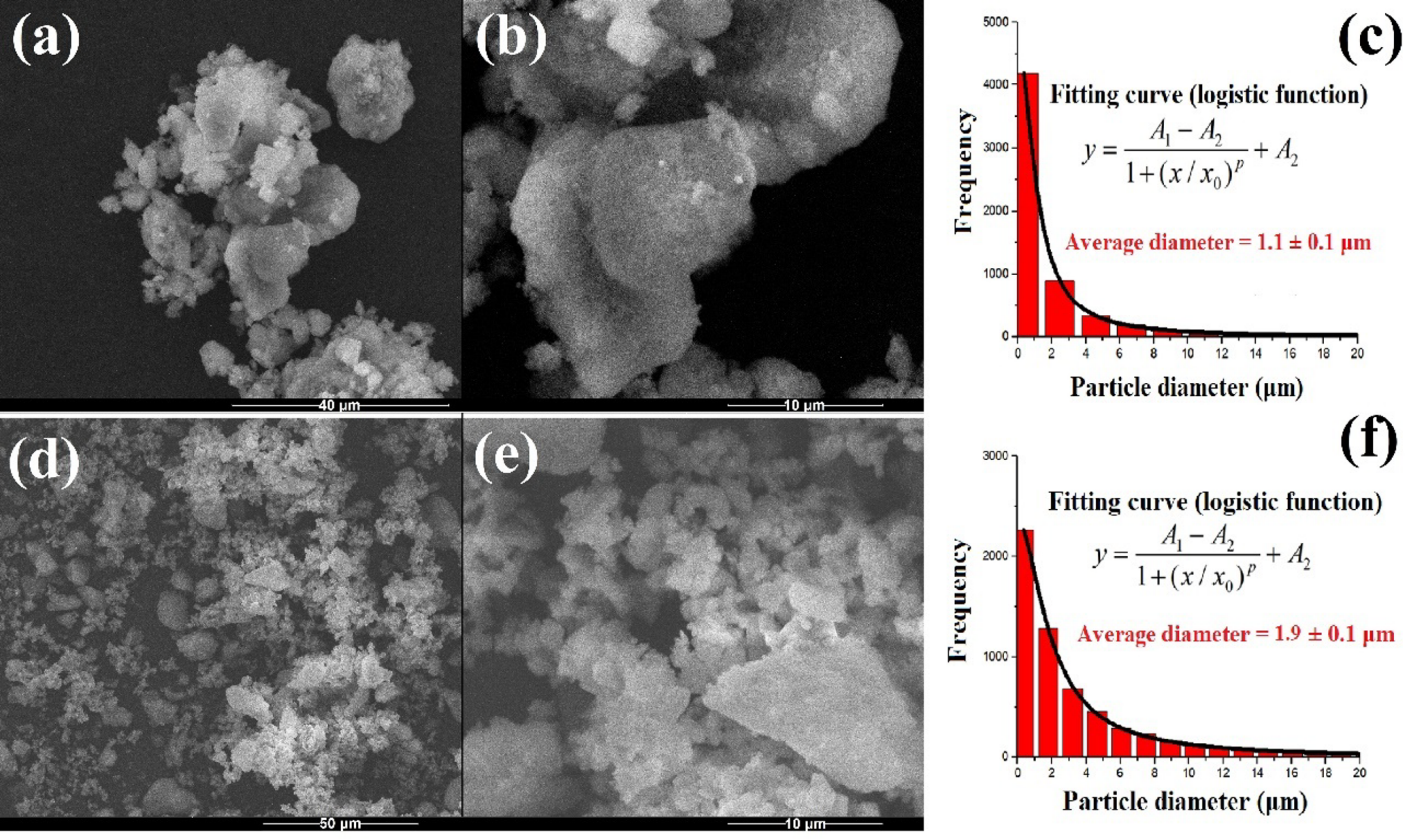
SEM micrographs of (a,b) Ag/SiO_2_ and (c,d) Ag/SiO_2_/ZPT/TCS powders, and their diameter distribution (e,f).

#### 3.1.2 Transmission Electron Microscopy

The HRTEM images of Ag/SiO_2_ (Figure 3b,c) reveal that some silver nanoparticles are embedded within the silica (SiO_2_) nanofillers. However, individual and loose silver nanoparticles can also be observed (Figure 3a). The silica appears as nanostructured fractal networks, typical of pyrogenic silica powders. Elemental analysis results for the hybrid nanoparticle systems are presented in Figure 4. Figure 4a–e shows the EDS mapping of the Ag/SiO_2_ system, indicating that silver nanoparticles are uniformly distributed within the SiO_2_ clusters. Spherical Ag nanoparticles are also seen as scattered dark patches around the SiO_2_ nanofillers, with diameters ranging from 8 to 20 nm (Figure S3).

**Figure 3:**
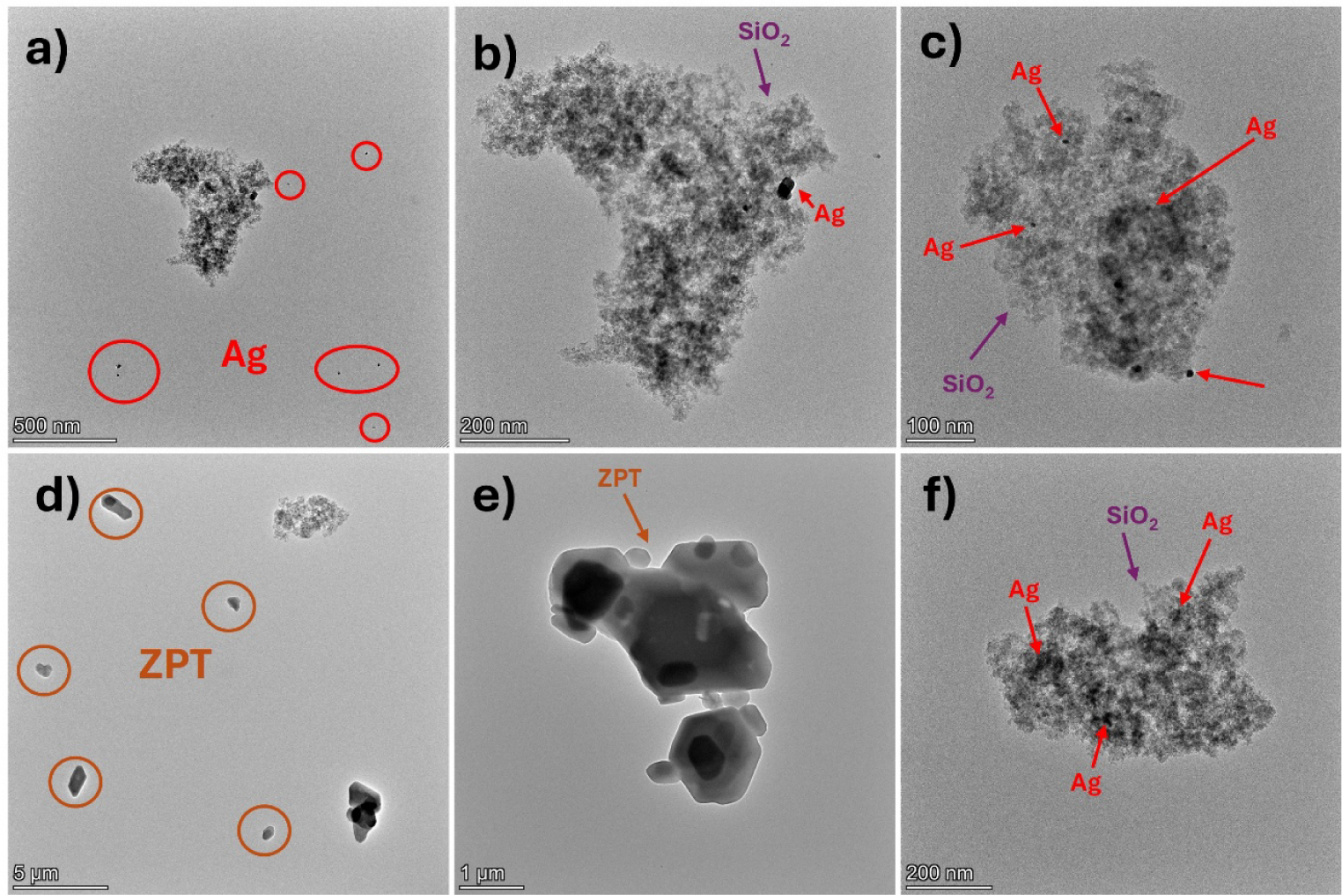
High-resolution transmission electron microscopy (HRTEM) images of (a–c) the Ag/SiO_2_ and (d–f) Ag/SiO_2_/ZPT/TCS nanoparticles.

**Figure 4:**
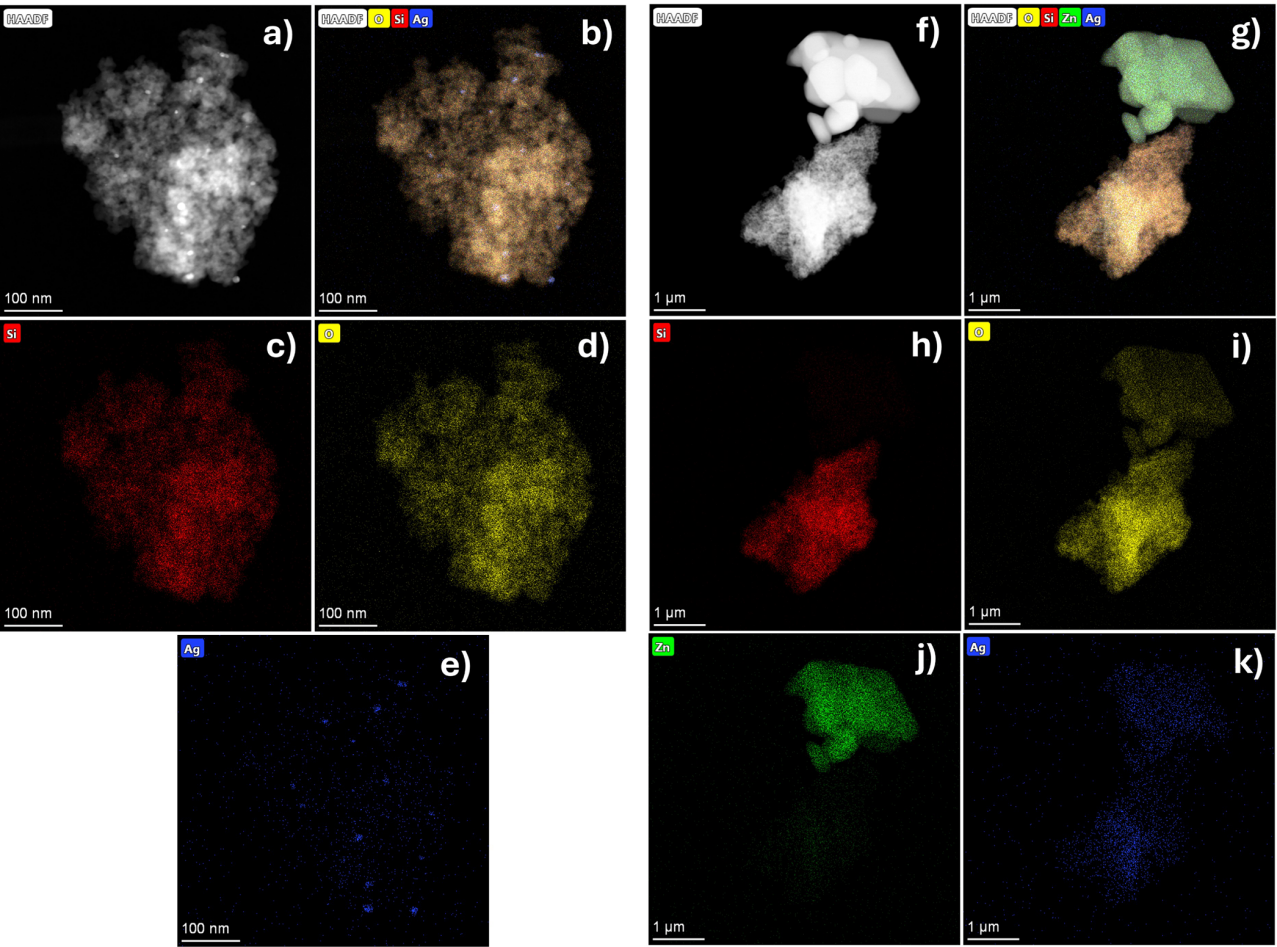
Energy dispersive spectroscopy (EDS) mapping of (a–c—Si, d—O, e—Ag) the Ag/SiO_2_ and (f–h—Si, i—O, j—Zn, k—Ag) Ag/SiO_2_/ZPT/TCS nanoparticles.

The Ag/SiO_2_/ZPT/TCS systems contain similar Ag/SiO_2_ nanoparticles, as shown in Figure 3f, with Ag nanoparticle sizes ranging from 10 to 46 nm (Figure S4c). In addition, TEM reveals the presence of another type of nano/microparticle (Figure 4d,e), characterized by faceted crystalline shapes and sizes between 1.8 and 2.4 μm (Figure S4a). According to the EDS mapping (Figure 4f–k), these particles contain at least zinc and oxygen, suggesting they correspond to the ZPT compound. No clear evidence of TCS was observed by TEM, likely due to the sample preparation process. The use of isopropanol may have dissolved and removed the compound during deposition.

#### 3.1.3 Thermogravimetric Analysis

The TGA and DTG curves of the powders are shown in Figure 5. DTG was calculated to help identify the thermal decomposition stages of the samples, and their respective characteristic temperatures. T_5%_, *T*_max_, and residual mass determined by DTG are detailed in Table 1. The Ag/SiO_2_ powder has a high moisture content (10%) due to the high affinity of water molecules with silica. As shown in Figure 5, after removing the moisture, this powder lost only 11% of its mass when heated to 500 °C, evidencing the absence of organic compounds. On the other hand, the Ag/SiO_2_/ZPT/TCS powder presents a more complex thermal decomposition process with three stages due to the loss of organic fractions on the surface of the silica particles, as seen in the DTG curve.

**Table 1:** Thermal Decomposition Onset Temperature (*T*_5%_), Maximum Thermal Decomposition Temperature (*T*_max_), and Residual Mass Results Obtained by TGA of the Powders

Powder	*T*_5%_ (°C)	*T*_max_ (°C)	Residual Mass at 500 °C (%)
Ag/SiO_2_	30	63 (loss of adsorbed moisture)	89
Ag/SiO_2_/ZPT/TCS	212	269, 338, and 561	38

**Figure 5:**
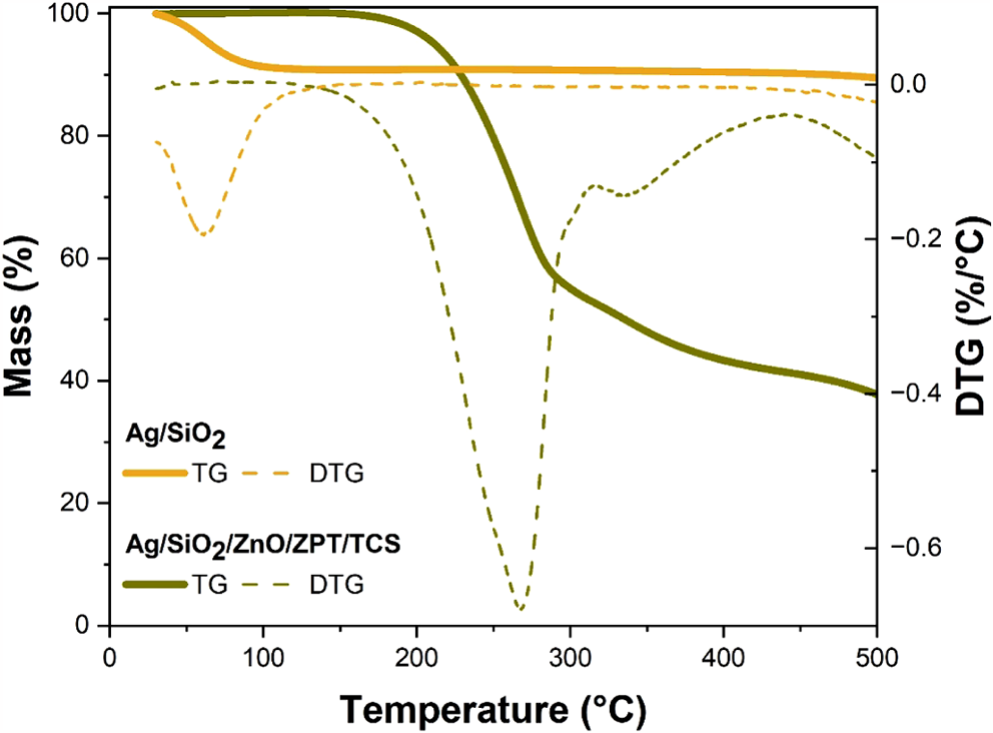
TGA and DTG curves for the Ag/SiO_2_ and Ag/SiO_2_/ZPT/TCS powders.

As reported in the literature, triclosan (TCS) totally degrades/evaporates between 160 and 340 °C and the DTG exhibits only one peak reported around 244 °C.^32^ Zinc pyrithione (ZPT) also starts to decompose in this range of temperature, and presents two degradation events, with the first one between 250 and 300 °C and another one between 300 and 370 °C,^23^ which correspond to ∼52% of weight loss up to 450 °C.^23^ Thus, the mass residue of ZPT at 450 °C corresponds to ∼48%. It is not possible to discriminate the content of each organic molecule due to the superposition of their degradation events.

Therefore, the weight loss corresponding to 62% that occurs up to 500 °C is related to the decomposition of triclosan and zinc pyrithione. Thus, the composition of Ag/SiO_2_/ZPT/TCS has a high content of organic compounds, at least more than 60%.

#### 3.1.4 FTIR Spectroscopy

Figure 6 shows that the Ag/SiO_2_ powder exhibits characteristic infrared absorption bands of SiO_2_:^33,34^ 795 cm^–1^ (Si–O–Si, symmetric stretching), 975 cm^–1^ (Si–O–H_2_O, bending), 1060 cm^–1^ (Si–O–Si, asymmetric stretching), and 3447 cm^–1^ (Si–OH and adsorbed H_2_O, stretching).^33,34^ The Ag/SiO_2_/ZPT/TCS powder presents the same characteristic silica signals, along with additional absorption bands attributed to organic groups from ZPT and TCS.

**Figure 6:**
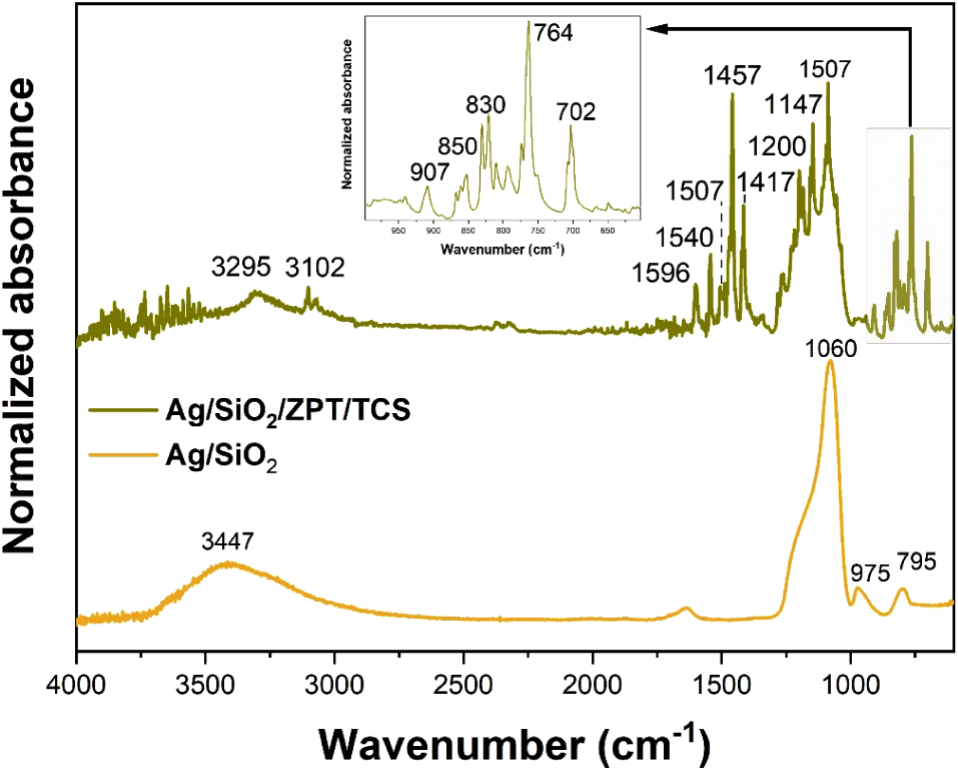
FTIR spectra of the Ag/SiO_2_ and Ag/SiO_2_/ZPT/TCS powders.

For the ZPT compound, a broad absorption at 3295 cm^–1^ corresponds to N–H stretching vibrations.^35^ The band at 3102 cm^–1^ is assigned to C–H aromatic stretching, while those at 1540 cm^–1^ and 1457 cm^–1^ correspond to C—C stretching vibrations of the aromatic ring.^22,35,36^ Additional bands at 1200 cm^–1^ and 1147 cm^–1^ are associated with C–O stretching and N–O stretching, respectively. A signal at 830 cm^–1^ corresponds to N–O bending vibrations, while the band at 702 cm^–1^ is attributed to C–S stretching.^22,36^ The absorption at 765 cm^–1^ is related to the out-of-plane deformation of —C–H bonds from the pyrithione ring structure.^37,38^ Regarding the TCS component, absorption peaks at 1598, 1507, and 1417 cm^–1^ correspond to C–C stretching within the benzene ring.^32,39,40^ Additional bands between 900 and 750 cm^–1^ are attributed to in-plane and out-of-plane bending modes of aromatic C–H bonds.^26,32^ Peaks at approximately 850 cm^–1^ and 830 cm^–1^ are associated with out-of-plane bending of aromatic C–H bonds and possible C–Cl wagging vibrations, characteristic of substituted benzene rings such as triclosan.^40^ These spectral features, often modified by interactions with inorganic phases such as SiO_2_, ZPT, or Ag, confirm the incorporation of triclosan within the hybrid material structure. The strong characteristics bands of ZPT and TCS confirm the high organic content of the Ag/SiO_2_/ZPT/TCS powder.

#### 3.1.5 ICP-OES

According to ICP-OES measurements, Ag/SiO_2_ and *Ag/SiO*_*2*_ */ZPT/TCS* powders contain 1.27 ± 0.13 and 0.77 ± 0.08 mg of silver per gram of powder, respectively, so Ag/SiO_2_ has a higher content of Ag (Table 2). The data evidence 0.31 ± 0.03 mg of zinc ions per gram of *Ag/SiO*_*2*_ */ZPT/TCS* powder which corresponds to 1.55 mg of ZPT per gram of the hybrid powdered compound.

**Table 2:** Chemical Element Content (mg/g , mg of Element per Gram of Sample) in the Powder Determined by ICP-OES

Chemical element	Ag/SiO_2_ powder	Ag/SiO_2_/ZPT/TCS powder
Ag	1.27 ± 0.13	0.77 ± 0.08
Zn	Not detected	0.31 ± 0.03

### 3.2 PVC-Based Composites

#### 3.2.1 Scanning Electron Microscopy (SEM)

The PVC compound utilized in this work is a commercial product containing calcium carbonate (CaCO_3_) and titanium dioxide (TiO_2_) microparticles dispersed in the polymeric matrix,^16^ as seen in SEM images in Figure 7. CaCO_3_ is utilized industrially as a filler to reduce the cost of the polymer.^41^ At the same time, CaCO_3_ reacts with hydrogen chloride (HCl), contributing to avoiding the autocatalytic degradation of PVC.^42^ TiO_2_ is applied as a white pigment and UV-blocking additive to protect different polymers against photodegradation caused by prolonged UV exposition.^43^ In the case of PVC, these microparticles can avoid diffusion and migration of the plasticizer to the PVC surface and external environment. The loss of plasticizer causes several changes in the mechanical performance of PVC products over time, compromising their performance as the plasticizer is leached.^44^

**Figure 7:**
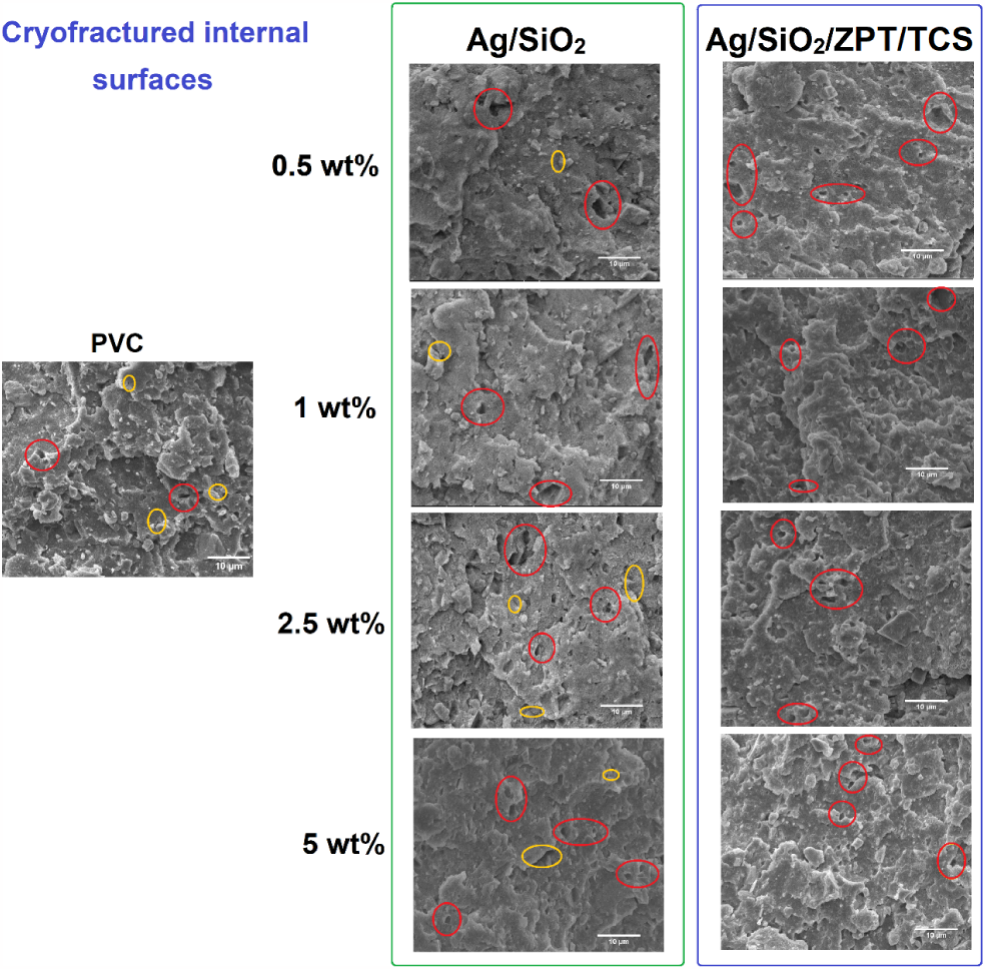
SEM images of the PVC, PVC/*X*(Ag/SiO_2_), and PVC/*X*(Ag/SiO_2_/ZPT/TCS) samples, where *X* corresponds to the concentration of the particles (Ag/SiO_2_ or Ag/SiO_2_/ZPT/TCS). Images were obtained from cryofractured internal surfaces of the samples. Different defects are highlighted in circles: cavities (red) and interfacial voids (yellow).

The PVC cryofractured internal surface exhibits microcavities and interfacial voids. The microcavities are associated with the detachment of inorganic particles, indicating low adhesion between the particles and the polymeric matrix. The interfacial voids also corroborate this weak adhesion between the phases. The SEM image of the external surface of the PVC (Figure S5) evidence the presence of CaCO_3_ and TiO_2_ microparticles. However, the addition of Ag/SiO_2_ and Ag/SiO_2_/PZT/TCS does not significantly impact the surface of the PVC films.^45,46^

The SEM images in Figure 7 indicate that all samples display a brittle cryofracture, which is attributed to the restricted mobility of the PVC polymer chains, thereby hindering shear band formation during fracture. While the PVC/*X*(Ag/SiO_2_) composites display both cavities and interfacial voids, the PVC/*X*(Ag/SiO_2_/ZPT/TCS) samples display only cavities suggesting that the Ag/SiO_2_ particles are less adhered to the PVC matrix and easily detach from it during the fracturing process. Then the organic functionalization of the Ag/SiO_2_ in Ag/SiO_2_/ZPT/TCS particles can be responsible for the lack of adhesion between the inorganic part of the particle and the PVC matrix.

#### 3.2.2 UV–Vis Diffuse Reflectance Spectroscopy

The diffuse reflectance (R_d_) spectra of all PVC samples in Figure 8 present light dispersion at 490 nm, indicating a sudden increase of the absorptivity and refractive index of the PVC sample at this wavelength. The Reststrahlen effect must be occurring on PVC samples at 490 nm due to Fresnel reflectance overcoming the Kubelk–Munk reflectance mechanism.^47^ The high UV radiation absorption at 280 and 245 nm is due to π–π* electronic transitions in the PVC polymer chains.^48^ The intense light absorption from 450 nm and the maximum UV absorption at 350 nm can be attributed to the CaCO_3_/TiO_2_ hybrid microparticles, which are low-cost commercial pigments available in the market applied as an alternative material to avoid the high price of TiO_2_ pigment, which is more expensive than CaCO_3_ powder.^49^

**Figure 8:**
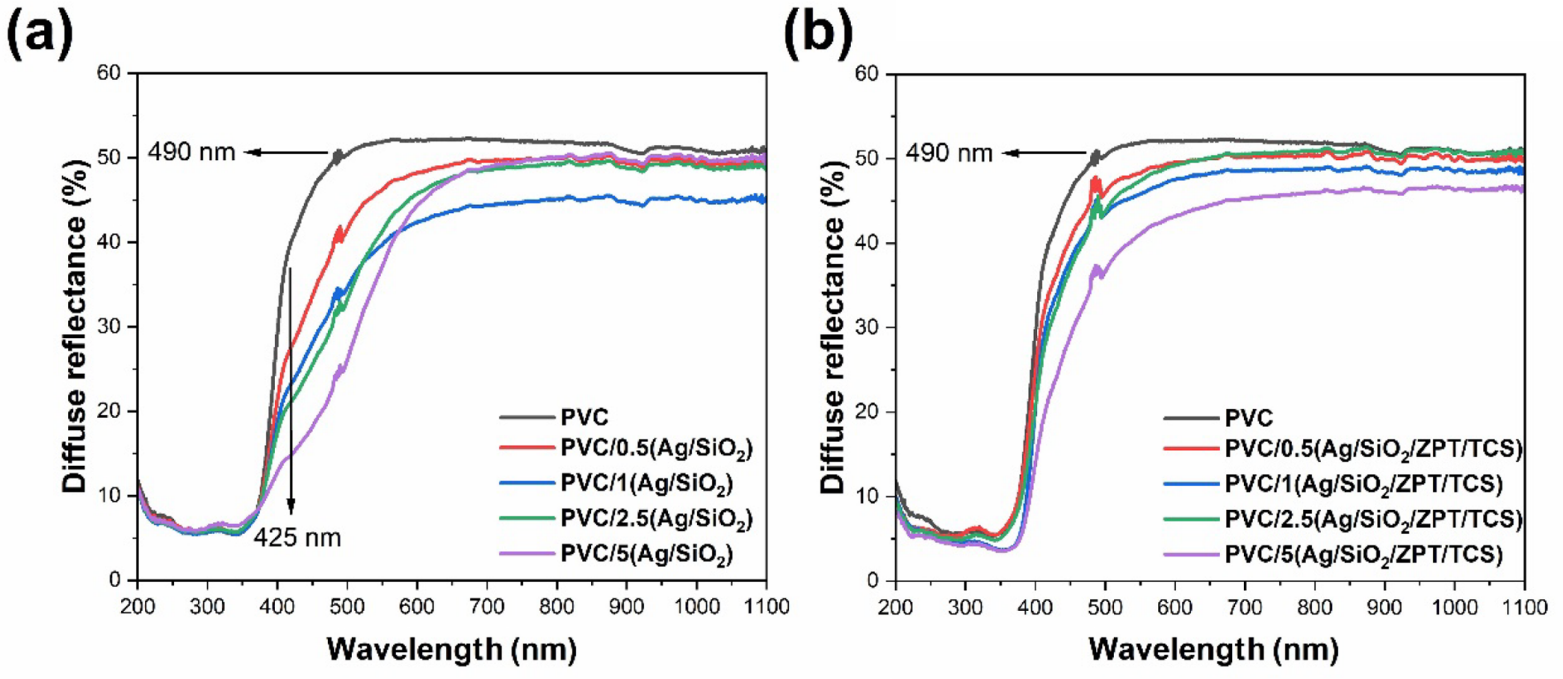
Diffuse reflectance (*R*_d_) spectra of the PVC, (a) PVC/*X*(Ag/SiO_2_), and (b) PVC/*X*(Ag/SiO_2_/ZPT/TCS) samples, where *X* corresponds to the concentration of the particles (Ag/SiO_2_ or Ag/SiO_2_/ZPT/TCS).

The diffuse reflectance of PVC in the range of 420–800 nm decreases slightly with higher Ag/SiO_2_ and Ag/SiO_2_/ZPT/TCS loadings, indicating increased photon absorption. The red-shifted broadening suggests defect-induced electronic levels, while the direct bandgap remains ≈3.0 eV and is unaffected by the additives.^48^

Tauc’s plots in Figure S6 of the (Kubelka–Munk) reveal direct $\left(E_{g}^{d}\right)$ and indirect bandgaps $\left(E_{g}^{i}\right)$ below those of neat PVC (4.2–4.3 eV) as listed in Table 3, with $E_{g}^{d}$ ≈ 3.0 eV—close to anatase TiO_2_ (3.3 eV) and above rutile (2.9 eV) that are typical additives in polymers.^50^ $E_{g}^{i}$ values range from 1.9 to 4.0 eV, below the $E_{g}^{i}$ values typical for CaCO_3_ (5.8 eV).^51^ Direct transitions originating from Ag/SiO_2_ can lower $E_{g}^{i}$ from 3.0 to 1.9 eV due to enhancement of phonon-assisted transitions via defect states.^29^

**Table 3:**
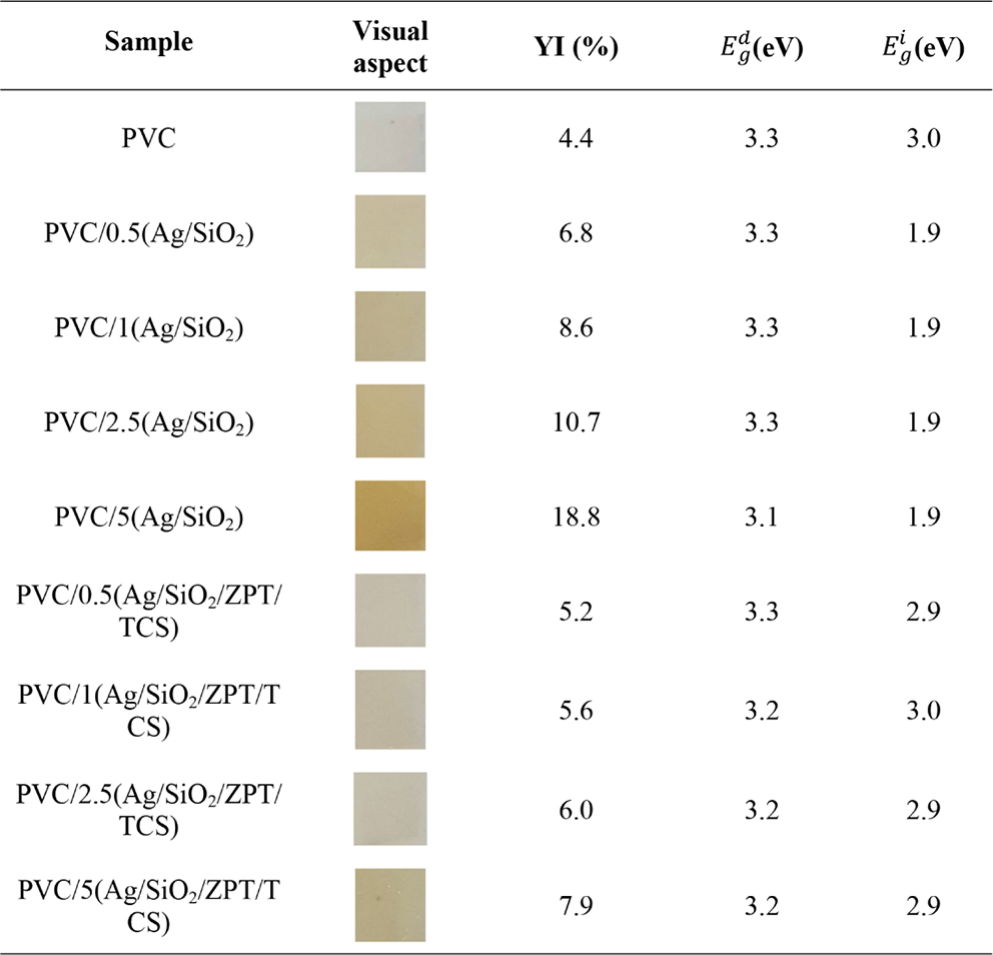
Visual Aspect, Yellowness Index (YI), Direct $\left(E_{g}^{d}\right)$ , and Indirect $\left(E_{g}^{i}\right)$ Optical Bandgaps of the PVC Samples

The yellowness index (YI) of the PVC rises as Ag/SiO_2_ and Ag/SiO_2_/ZPT/TCS contents increase. This can be due to increased degradation of the PVC matrix during thermal processing or by the coloration given by the additives. PVC thermo-oxidatively degrades via zipper dehydrochlorination, leading to conjugated double bonds.^52^ As the number of conjugated double bond increases, the polymer undergoes a color change, shifting progressively from white to yellow, orange, red, brown, and eventually black.^53^ Although ZPT has been reported to affect the heat stability of flexible PVC leading to a change of color, particularly in formulations prepared in internal mixers,^54^ herein, the YI values suggest that the Ag/SiO_2_/ZPT/TCS powder causes less pronounced color changes compared to Ag/SiO_2_, as seen in the photographs of the samples in Table 3. The presence of triclosan, which is a white powder, may also contribute positively to maintaining the original color of PVC/Ag/SiO_2_/ZPT/TCS.

#### 3.2.3 Mechanical Properties

Tukey’s test indicates that both Ag/SiO_2_ and Ag/SiO_2_/ZPT/TCS antimicrobial particles significantly affect Young’s modulus (E), impact strength, and ultimate tensile strength (σ_max_) of the PVC in all the compositions evaluated. The E, σ_max_, and impact strength values are detailed in Table 4.

**Table 4:** Young’s Modulus (E), Impact Strength, and Ultimate Tensile Strength (σ_max_) of the PVC Samples

Sample	E (GPa)	σ_max_ (MPa)	Impact strength (J m^–1^)
PVC	2.1 ± 0.3	45.1 ± 4.9	100.0 ± 8.9
PVC/0.5(Ag/SiO_2_)	2.3 ± 0.2	42.6 ± 3.6	100.2 ± 16.3
PVC/1(Ag/SiO_2_)	2.3 ± 0.1	37.8 ± 1.9	91.5 ± 16.8
PVC/2.5(Ag/SiO_2_)	2.5 ± 0.3	45.1 ± 5.3	100.6 ± 27.7
PVC/5(Ag/SiO_2_)	2.4 ± 0.4	46.7 ± 2.6	71.5 ± 6.8
PVC/0.5(Ag/SiO_2_/ZPT/TCS)	1.6 ± 0.1	39.0 ± 3.1	118.6 ± 40.5
PVC/1(Ag/SiO_2_/ZPT/TCS)	1.7 ± 0.1	38.8 ± 3.4	133.1 ± 30.8
PVC/2.5(Ag/SiO_2_/ZPT/TCS)	1.8 ± 0.2	38.8 ± 2.8	92.2 ± 24.6
PVC/5(Ag/SiO_2_/ZPT/TCS)	1.7 ± 0.1	39.9 ± 2.1	107.5 ± 17.2

PVC presents a Young’s modulus of 2.1 ± 0.3 GPa, which increases to 2.3–2.4 GPa with the addition of Ag/SiO_2_ and decreases to 1.6–1.8 GPa upon the introduction of Ag/SiO_2_/ZPT/TCS. The incorporation of silica nanoparticles from the Ag/SiO_2_ system slightly increases the tensile modulus of the PVC compounds. In contrast, the Ag/SiO_2_/ZPT/TCS hybrid system reduces the modulus, likely due to the presence of the TCS organic compound, which may exert a plasticizing effect on the PVC matrix. Similarly, σ_max_ decreases from 45.1 ± 4.9 MPa to 38–40 MPa with the addition of Ag/SiO_2_/ZPT/TCS particles, also possibly due to a reduction in the polymer’s cohesive strength resulting from the plasticizing effect of the organic compounds. The Ag/SiO_2_ system, however, does not significantly affect the tensile strength of the PVC compound.

Regarding impact strength, Ag/SiO_2_ particles have little influence, except at the highest concentration (5%), where the likely formation of coarse SiO_2_ aggregates may have acted as stress concentrators, leading to increased brittleness. On the other hand, the Ag/SiO_2_/ZPT/TCS hybrid nanoparticles tend to enhance the impact strength in most samples, despite the large standard deviation observed. The organic compounds probably play a secondary role acting as toughening agents on PVC, due to a plasticizing/lubricating action.^55,56^

If analyzed more generally, the comparison of properties of the polymeric systems via an interactive method between pairs by ANOVA two-way (Table S3) indicates that the Ag/SiO_2_ affects only the modulus significantly. In contrast, Ag/SiO_2_/ZPT/TCS particles influence the modulus and tensile strength.

Values are presented as mean ± standard deviation (SD).

#### 3.2.4 Thermogravimetric Analysis (TGA)

The TGA curves of the PVC samples are shown in Figure 9. All samples present two decomposition stages associated with the dehydrochlorination (250–350 °C) and decomposition of the polyene sequences from the previous PVC dehydrochlorination step (420–550 °C).^57,58^ PVC dehydrochlorination involves around 50 wt % of mass loss, while the thermal decomposition of the polyenes can reach 20 wt %, being slightly reduced with the increase of the Ag/SiO_2_ or Ag/SiO_2_/ZPT/TCS contents in the PVC matrix. Carbonaceous residues and inorganic additives observed in the PVC by SEM correspond to 30 wt % of the PVC samples at 600 °C.

**Figure 9:**
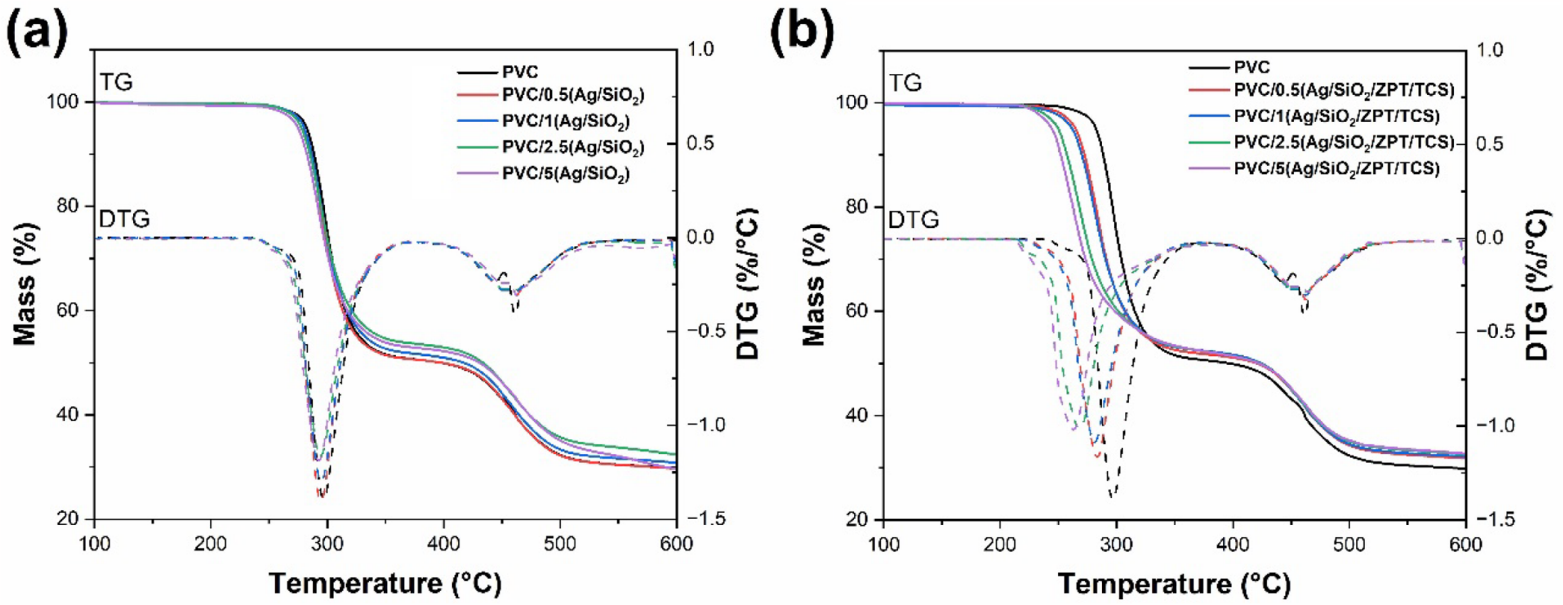
TGA and DTG curves of (a) PVC/*X*(Ag/SiO_2_) and (b) PVC/*X*(Ag/SiO_2_/ZPT/TCS) samples, where *X* corresponds to the concentration of the particles (Ag/SiO_2_ or Ag/SiO_2_/ZPT/TCS).

The onset thermal decomposition temperature (*T*_*onset*_) of PVC is 281 ± 5 °C, while its temperatures at maximum thermal decomposition rate (*T_max_*) are 302 °C (first thermal decomposition step) and 460 °C (second thermal decomposition step), as detailed in Table 5. The *T_onset_* is reduced with the increase of Ag/SiO_2_ and Ag/SiO_2_/ZPT/TCS concentrations, indicating that these antimicrobial additives compromise the thermal stability of the PVC matrix, that corroborates the YI data from UV–vis spectra. Ag/SiO_2_/ZPT/TCS particles cause the highest reduction of the composites *T*_*onset*_, from 281 ± 5 °C for pure PVC to 241 ± 5 °C for PVC/5­(Ag/SiO_2_/ZPT/TCS). These organic compounds accelerate PVC thermal degradation, as evidenced by the reduction in the *T_max_* associated with the PVC dehydrochlorination step when the Ag/SiO_2_/ZPT/TCS loading is increased. This effect is not observed with Ag/SiO_2_. The combination of ZPT and TCS with Ag/SiO_2_ introduce chlorine-rich organics and Zn^2+^, forming ZnCl_2_ under heat that catalyzes dehydrochlorination and chain unzipping. Triclosan decomposes into chlorine radicals and phenoxy species,^59^ accelerating chain scission and HCl release.^60^ Consequently, the *T*_*onset*_ decreases and the thermal degradation rate of PVC increases. However, the *T_max_* associated with the PVC second thermal decomposition step is not affected by either antimicrobial agent.

**Table 5:** *T*_*onset*_ and *T_max_* Temperatures from TGA and DTG Measurements

Sample	*T*_*onset*_ (°C)	*T_max_* (°C)
PVC	281 ± 5	297
		461
PVC/0.5(Ag/SiO_2_)	278 ± 5	295
		463
PVC/1(Ag/SiO_2_)	278 ± 5	395
		463
PVC/2.5(Ag/SiO_2_)	275 ± 5	294
		463
PVC/5(Ag/SiO_2_)	273 ± 5	293
		463
PVC/0.5(Ag/SiO_2_/ZPT/TCS)	261 ± 5	283
		463
PVC/1(Ag/SiO_2_/ZPT/TCS)	259 ± 5	281
		463
PVC/2.5(Ag/SiO_2_/ZPT/TCS)	246 ± 5	269
		464
PVC/5(Ag/SiO_2_/ZPT/TCS)	241 ± 5	263
		464

#### 3.2.5 Aqueous Release of Organic and Inorganic Species

The water extracts in contact with PVC/Ag/SiO_2_ exhibited UV–Vis spectra indistinguishable from the PVC control, with no localized surface plasmon resonance (LSPR) feature in the 390–430 nm range typically associated with dispersed Ag nanoparticles.^61^ Consistently, ICP-MS indicated only traces of Zn and ultratraces of Ag in PVC/Ag/SiO_2_ leachates – at or below the method detection limit (Table 6). Taken together, these observations are consistent with minimal release and, when release occurs, a predominance of soluble Ag species at levels below the UV–Vis detectability of nanoparticulate Ag.^62^

**Table 6:** Chemical Element Content (μg/L, μg of Element per Gram of Sample) in the 120 min Aliquots Analyzed by ICP-MS

Chemical element	Ag	Zn
PVC	Not detected	Not detected
PVC/0.5(Ag/SiO_2_)	0.14 ± 0.03	Not detected
PVC/1(Ag/SiO_2_)	0.29 ± 0.04	Not detected
PVC/2.5(Ag/SiO_2_)	0.25 ± 0.03	Not detected
PVC/5(Ag/SiO_2_)	1.05 ± 0.04	Not detected
PVC/0.5(Ag/SiO_2_/ZPT/TCS)	Not detected	Not detected
PVC/1(Ag/SiO_2_/ZPT/TCS)	Not detected	Not detected
PVC/2.5(Ag/SiO_2_/ZPT/TCS)	Not detected	15.68 ± 0.93
PVC/5(Ag/SiO_2_/ZPT/TCS)	Not detected	14.14 ± 0.64

In contrast, the UV–Vis spectra for the PVC/Ag/SiO_2_/ZPT/TCS system showed a clear time dependence, with absorbance increasing over the 30, 60, and 120 min intervals (Figure 10). This is consistent with a time-dependent lixiviation process, driven primarily by triclosan, which produced a well-defined band centered at ∼285 nm with a shoulder at ∼232–235 nm, in line with TCS in aqueous media.^63,64^ For the 2.5% and 5% samples, the UV–Vis absorbance also rises from ∼340 nm onward. ICP-MS detected Zn in these extracts (∼15 ± 1 μg L^–1^), whereas Ag remained below the method detection limit. ZPT-related signals were only at trace levels in some extracts and did not yield distinct UV–Vis features. This pattern suggests preferential leaching of TCS and Zn at trace levels at higher loadings, whereas Ag remains largely retained within the matrix or present in forms not solubilized under the extraction conditions.

**Figure 10:**
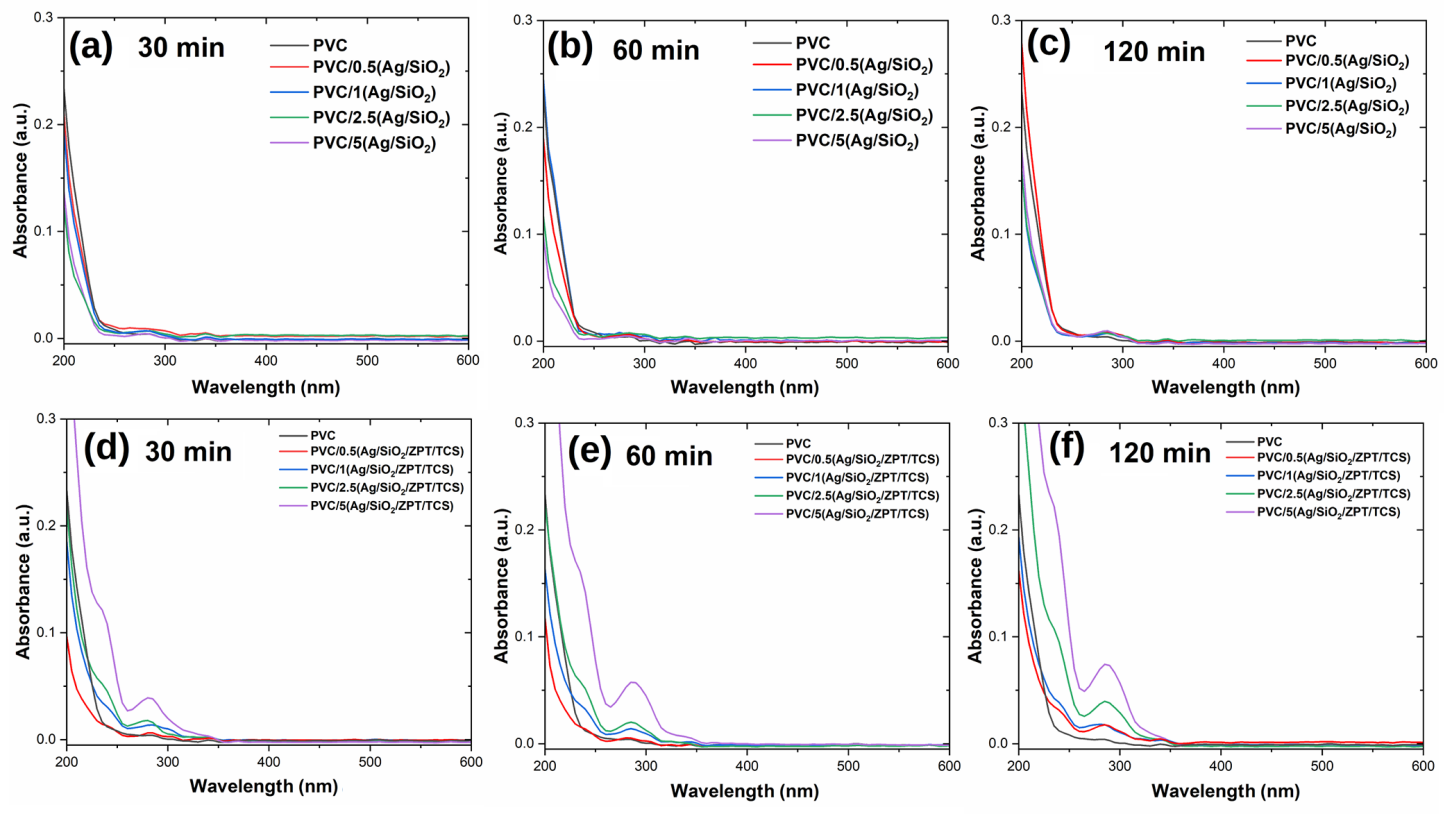
UV–vis absorption spectra of the aqueous extracts from PVC/X­(Ag/SiO_2_) at contact times of (a) 30 min, (b) 60 min, and (c) 120 min; and PVC/X­(Ag/SiO_2_/ZPT/TCS) at (d) 30 min, (e) 60 min, and (f) 120 min.

#### 3.2.6 Antiviral and Cell Viability Assessment

The antiviral assay was performed at different incubation times (30, 60, and 120 min), for pure PVC, PVC/X­(Ag/SiO_2_), and PVC/X­(Ag/SiO_2_/ZPT/TCS). A clear time-dependent relationship was observed, where longer contact times resulted in greater percentages of viral inactivation as measured through cell viability (Figure 11). The progression of antiviral activity with time suggests a kinetic process dependent on the interaction time between the viral particles and the nanocomposites.

**Figure 11:**
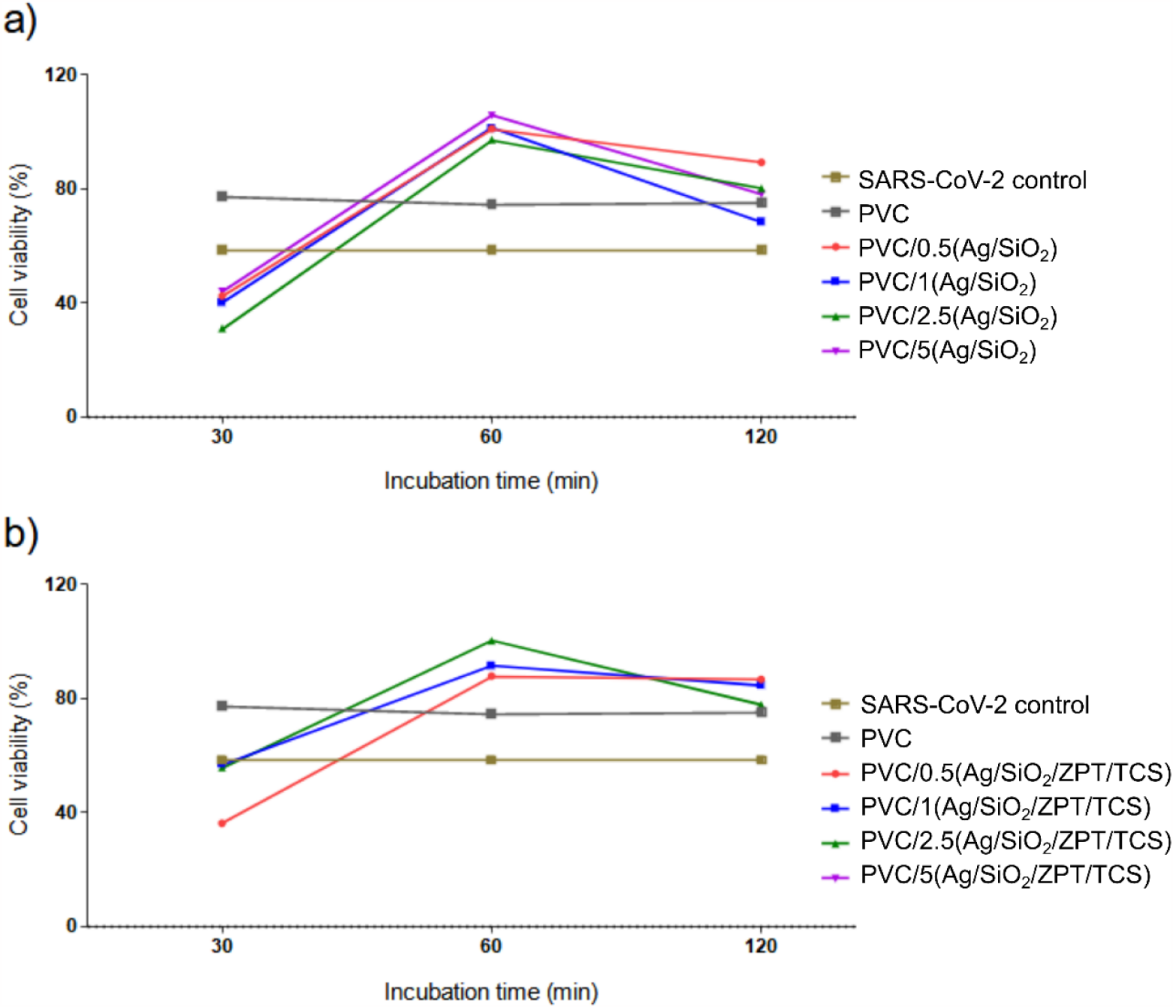
Cell viability within different incubation times at the surfaces of PVC nanocomposites: measured by the MTT reduction assay using absorption measurements at 570 nm (SkanIt Software 2.4.5, Varioskan Flash, Thermo Fisher, USA). (a) PVC, PVC/X­(Ag/SiO_2_), and (b) PVC/X­(Ag/SiO_2_/ZPT/TCS) nanocomposites.

At the contact time of 120 min, most PVC nanocomposite samples maintained adequate cell viability compared to cellular controls, with one notable exception. The sample PVC/5­(Ag/SiO_2_/ZPT/TCS) exhibited reduced cell viability that prevented reliable assessment of its antiviral potential, indicating potential cytotoxic effects at this concentration (Figure 12).

**Figure 12:**
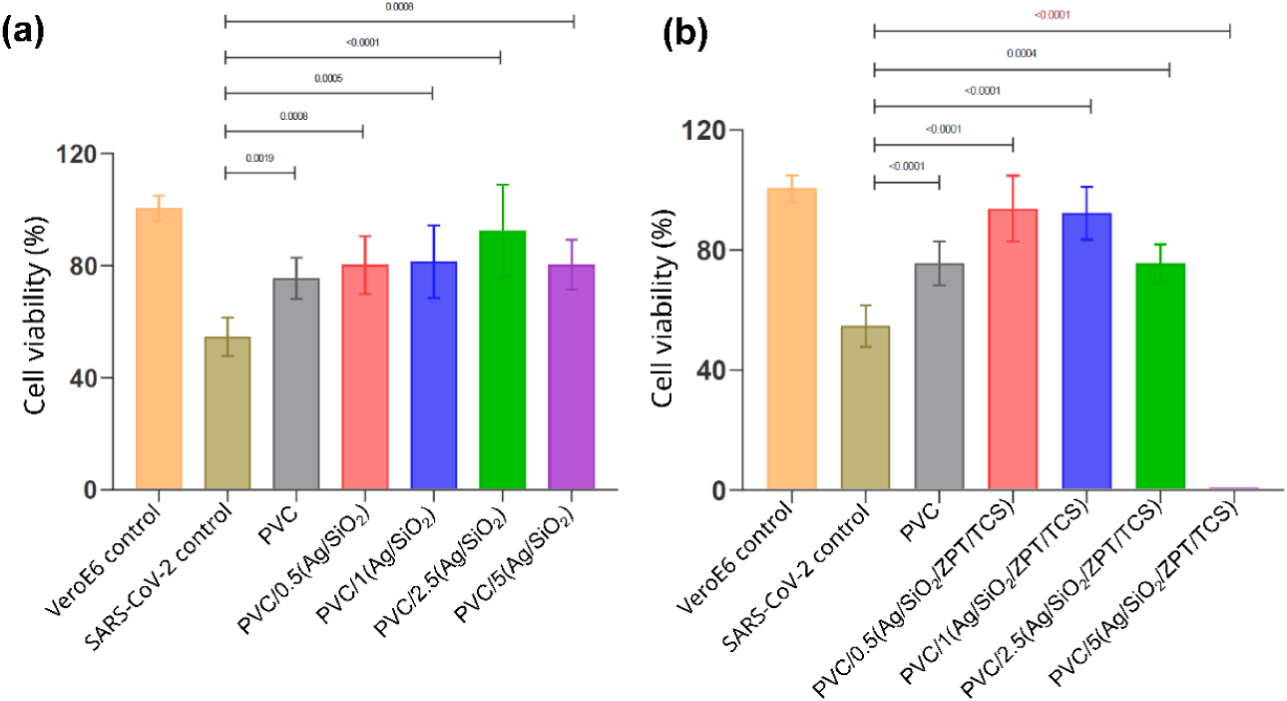
Cell viability at the incubation time of 120 min of PVC nanocomposites: (a) PVC PVC/X­(Ag/SiO_2_) (b) PVC/X­(Ag/SiO_2_/ZPT/TCS) nanocomposites. Data presented as average ± standard deviation (SD). ANOVA one-way analysis was applied, followed by Dunn’s variance test— horizontal bars indicate significant differences between the data.

Among the nanocomposites with Ag/SiO_2_, only PVC/2.5­(Ag/SiO_2_) achieved the virucidal threshold of ≥99.99% inactivation (4-log reduction). In contrast, the hybrid Ag/SiO_2_/ZPT/TCS systems demonstrated enhanced virucidal activity at lower loadings. Both PVC/0.5­(Ag/SiO_2_/ZPT/TCS) and PVC/1.0­(Ag/SiO_2_/ZPT/TCS) achieved 99.99% inactivation and were classified as virucidal (Table 7).

**Table 7:** Antiviral Activity Results from the PVC and the Hybrid Nanocomposites

Sample	Log reduction	Inactivation percentage (%)	Activity
PVC	2	99	Not virucidal
PVC/0.5(Ag/SiO_2_)	3	99.9	Not virucidal
PVC/1(Ag/SiO_2_)	3	99.9	Not virucidal
PVC/2.5(Ag/SiO_2_)	4	99.99	Virucidal
PVC/5(Ag/SiO_2_)	3	99.9	Not virucidal
PVC/0.5(Ag/SiO_2_/ZPT/TCS)	4	99.99	Virucidal
PVC/1(Ag/SiO_2_/ZPT/TCS)	4	99.99	Virucidal
PVC/2.5(Ag/SiO_2_/ZPT/TCS)	2	99	Not virucidal
PVC/5(Ag/SiO_2_/ZPT/TCS)	-	-	Not evaluated

According to the release tests, traces of ZPT and ultratrace levels of Ag^+^ are released from the nanocomposites containing Ag/SiO_2_ and Ag/SiO_2_/ZPT/TCS. Moreover, triclosan is progressively released by the PVC/*X*(Ag/SiO_2_/ZPT/TCS) nanocomposites. Consequently, the virucidal effect observed is not due to the release of Ag^+^ ions, unlike in other systems,^65^ but must be related to virus inactivation upon contact with the nanoparticles of Ag^0^ on the nanocomposites surface, by disrupting viral integrity.^17,66^

The performance of the nanocomposites containing Ag/SiO_2_/ZPT/TCS is attributed to a combined action of silver nanoparticles and TCS.

The presence of TCS introduces complementary antiviral mechanisms. TCS disrupts viral lipid membranes, denatures viral envelope proteins, and contributes to oxidative damage, particularly when immobilized in polymeric matrices.^24,25^ This combined action enables effective viral inactivation at reduced filler loadings (0.5 and 1 wt %), potentially minimizing cytotoxic effects while maintaining virucidal efficacy. The progressive release of TCS contribute to the greater percentages of viral inactivation obtained at longer times. However, ZPT did not contribute to the antiviral activity as evidenced by the traces of Zn detected in the release medium.

The sample with 5 wt % of Ag/SiO_2_ and 2.5 wt % Ag/SiO_2_/ZPT/TCS unexpectedly presented reduced performance (99.9% and 99%, respectively) compared to their counterparts that contained lower amount of particles and exhibited virucidal property, which may result from excessive filler content causing nanoparticle aggregation reducing the area of contact with the virus, or impaired TCS diffusion.^15,67,68^ Such nonlinear behavior is consistent with prior findings on AgNP- and TCS-based systems, where optimized loading ensures maximum surface activity without physical obstruction or mass-transfer limitations.^24,25,68^

The sample with 5 wt % Ag/SiO_2_/ZPT/TCS demonstrated a reduction in cell viability indicative of cytotoxic effects *in vitro*, which hindered the reliable evaluation of its antiviral activity. Despite the presence of antiviral agents, the compromised cellular viability limited the possibility of assessing and classifying its virucidal potential accurately.

Overall, the antiviral response of PVC nanocomposites is strongly dependent on both the composition and concentration of the embedded nanophases. While Ag/SiO_2_ systems rely primarily on the AgNPs activity, the Ag/SiO_2_/ZPT/TCS formulations benefit from multitarget mechanisms involving silver and triclosan, that act together to inactivate SARS-CoV-2 even at lower filler levels.

Although our antiviral assessments lasted up to 120 min of incubation, recent studies demonstrated that SARS-CoV-2 can persist on surfaces such as plastic and stainless steel for 6 h to several days under ambient conditions, depending on the material and environmental factors.^69^ Therefore, while rapid virucidal activity within the initial 2 h is critical for reducing early transmission risks, future studies should include longer incubation times to better mimic real-world scenarios of viral persistence and confirm the long-term antiviral efficacy of the tested materials, inclusively under environmental exposure and mechanical stress.

## 4 Conclusions

The development of antimicrobial polymeric materials has become increasingly vital for biomedical and public health applications, particularly in response to viral pandemics such as COVID-19. In this work, poly­(vinyl chloride) (PVC) nanocomposites were engineered with antimicrobial hybrid nanoparticles composed of silver–silica (Ag/SiO_2_) and silver–silica functionalized with zinc pyrithione and triclosan (Ag/SiO_2_/ZPT/TCS), using a melt-compounding technique.

The incorporation of Ag/SiO_2_ increased the polymer’s Young’s modulus, while Ag/SiO_2_/ZPT/TCS composites decreased the stiffness due to a probable plasticizing/lubricant effect caused by the organic compounds present in the hybrid system. These compounds also increased the impact strength, but reduced the polymer’s thermal stability. Concerning the optical properties, Ag/SiO_2_ substantially reduced the indirect optical bandgap $\left(E_{g}^{i}\right)$ of the PVC from 3.0 to 1.9 eV, but Ag/SiO_2_/ZPT/TCS had no significant effect. The yellowness index of Ag/SiO_2_ composite rose with particle concentration, whereas this tendency was limited for Ag/SiO_2_/ZPT/TCS, due to the white color of the organic fraction and to their plasticizing effect, limiting PVC degradation during processing.

Remarkably, both systems demonstrated virucidal activity against SARS-CoV-2. While 2.5 wt % of Ag/SiO_2_ was required to reach 99.99% of viral inactivation, the Ag/SiO_2_/ZPT/TCS system achieved comparable performance at only 0.5–1.0 wt %, indicating a combined antiviral effect between Ag nanoparticles and released TCS. However, the highest loading of the hybrid system (5 wt %) resulted in cytotoxicity, emphasizing the importance of dose optimization.

This study demonstrates the potential of functional hybrid powders as effective antimicrobial fillers for PVC-based materials, offering tunable performance through tailored filler chemistry and loading. The combined action of inorganic and organic compounds showed superior antiviral activity, although both systems investigated are promising for producing materials with antiviral/antimicrobial properties. The Ag/SiO_2_ system requires higher concentrations to achieve viral inactivation but has little impact on the mechanical performance of PVC. Conversely, the Ag/SiO_2_/ZPT/TCS system inactivated the virus at lower concentrations, offering potential cost-effectiveness, but it reduces of the stiffness of the PVC compound. For applications where this mechanical trade-off is acceptable, the hybrid inorganic/organic system is likely the more suitable option. Importantly, both additives are commercially available and can be readily incorporated into PVC and other polymer formulations, most practically in the form of masterbatches, enabling straightforward integration into existing processing lines. These findings support the development of scalable functional biomaterials using conventional processing techniques, with potential applications in biomedical devices and hospital infrastructure, such as handrails.

## Supplementary Material



## Notes

ICP-OES and ICP-MS operating parameters; SEM and diameter distribution fitting of Ag/SiO_2_ and Ag/SiO_2_/ZPT/TCS; TEM and particle average diameter of the Ag in Ag/SiO_2_ and Ag/SiO_2_/ZPT/TCS; SEM analysis of the PVC composites; UV–vis diffuse reflectance spectroscopy; mechanical properties (PDF)
