# Supplementary material for: Hybrid Silver–Silica and Organic Biocide Systems in PVC: Enhanced Antiviral Performance against SARS-CoV‑2
Source: ACS Appl Mater Interfaces. 2025 Sep 24;17(40):56439–53. doi: 10.1021/acsami.5c12270 (PMC12516676; doi:10.1021/acsami.5c12270)
Supplement: Supplementary file 1 [file am5c12270_si_001.pdf]

Supporting Information

Hybrid Silver–Silica and Organic Biocide Systems in PVC: Enhanced Antiviral Performance Against SARS-CoV-2

Daniel J. da Silva[1], Guilherme B. Gramcianinov[1], Vanessa B. Malaquias[2], Pamela Z. Jorge[1], Cecilia Gonsales[1], Eduardo W. A. Pereira[2], Leice G. Amurin[1], Mário H. Hirata[2], Caroline C. Augusto[3], Bruno L. Batista[3], Beatriz B. Alves[4], Luciano A. Bueno[1], Danilo J. Carastan[1*], Mathilde Champeau[1*]

[1] Center for Engineering, Modeling and Applied Social Sciences (CECS), Federal University of ABC (UFABC), Av. dos Estados, 5001, Santo André, CEP 09210-580, SP, Brazil.

[2] Department of Clinical and Toxicological Analysis, Faculty of Pharmaceutical Sciences, University of São Paulo, Av. Professor Lineu Prestes, 580, São Paulo, CEP 05508-900, SP, Brazil.

[3] Center for Natural and Human Sciences (CCNH), Federal University of ABC (UFABC), Av. dos Estados, 5001, Santo André, CEP 09210-580, SP, Brazil.

[4] BRGoods Indústria e Comércio de Produtos Hospitalares, R. Antônio Barnabé, 1398, Indaiatuba, CEP: 13347-340, SP, Brazil

E-mail addresses: danilo.carastan@ufabc.edu.br (D.J. Carastan), mathilde.champeau@ufabc.edu.br (M. Champeau)

ICP-OES operating parameters

Table S1. ICP-OES operating parameters for silver and zinc quantification.

**Plasma Parameter**	**Condition**
Radiofrequency power	1 kW
Plasma gas flow	15 L min^{-1}
Auxiliary gas flow	1.5 L min^{-1}
Nebulization pressure	200 kPa
Replicate read time	5
Stabilization delay	15 s
Sample uptake delay	15 s
Pump rate	15 rpm
Rinse time	10 s
**Spectral line**	**Condition**
Ag	328.068 nm
Cu	327.395 nm
Zn	213.857 nm

SEM and diameter distribution fitting of Ag/SiO_2 and Ag/SiO_2/ZPT/TCS

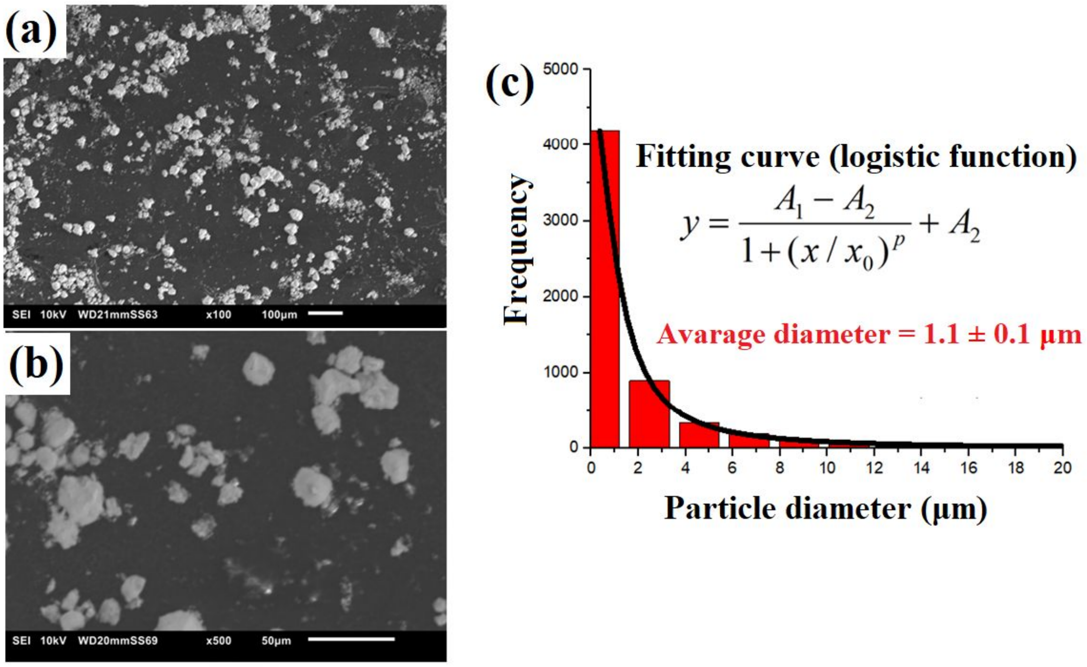

Figure S1. SEM micrographs (a, b), and particle diameter distribution (c) of the Ag/SiO_2 powder. Diameter destruction follows a logistic function (R^2 = 0.999). The logistic function (c): A_1 and A_2 correspond to the largest and smallest diameters of the particles identified in the electron micrograph; x_o is the mean particle diameter; x is the particle diameter; p is the exponential index of the logistic distribution for particle diameters.

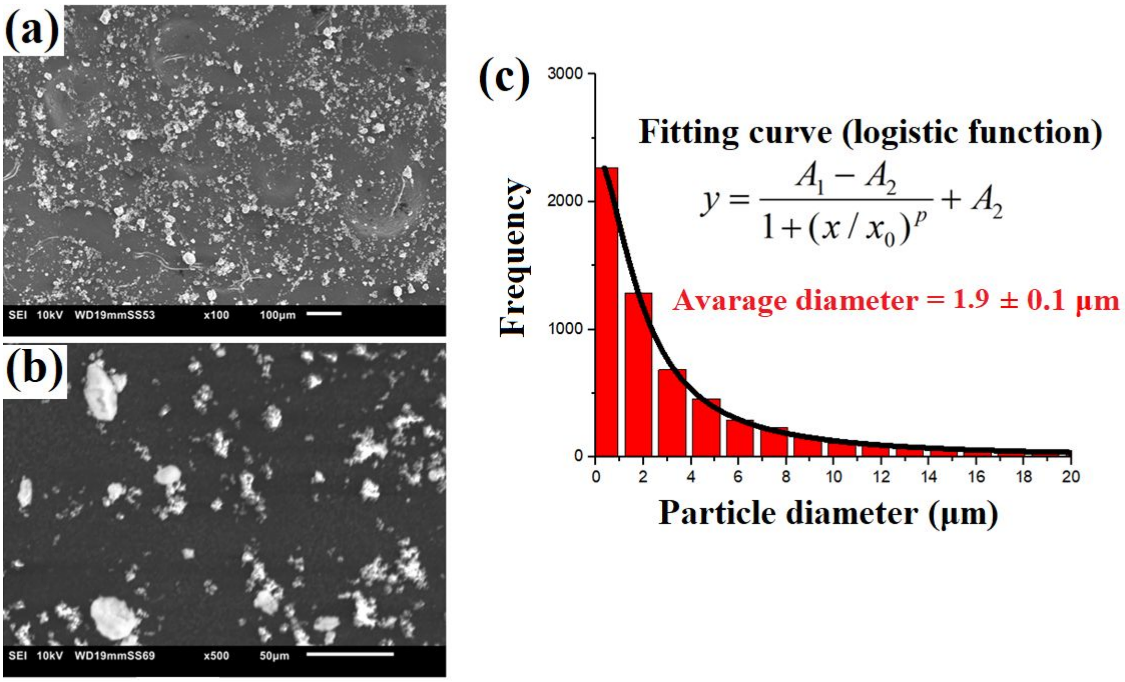

Figure S2. SEM micrographs (a, b) and particle diameter distribution (c) of the Ag/SiO_2/ZPT/TCS powder. Diameter destruction follows a logistic function (R^2 = 0.999). The logistic function (c): A_1 and A_2 correspond to the largest and smallest diameters of the particles identified in the electron micrograph; x_o is the mean particle diameter; x is the particle diameter; p is the exponential index of the logistic distribution for particle diameters.

Table S2. Parameters obtained by the logistic function fitting for the particle diameter distribution from SEM data. A_1 and A_2 correspond to the largest and smallest diameters of the particles in micrometers identified in the SEM images; x_o is the mean particle diameter; p is the exponential index of the logistic distribution for particle diameters.

Parameter	Ag/SiO_2	Ag/SiO_2/ZPT/TCS
A_1	4738.06 ± 13.29	2387.75 ± 8.57
A_2	-0.44 ± 0.22	-5.61 ± .91
x_o	1.11 ± 0.01	1.95 ± 0.01
p	1.81 ± 0.01	1.73 ± 0.01

TEM and particle average diameter of the Ag in Ag/SiO_2 and Ag/SiO_2/ZPT/TCS

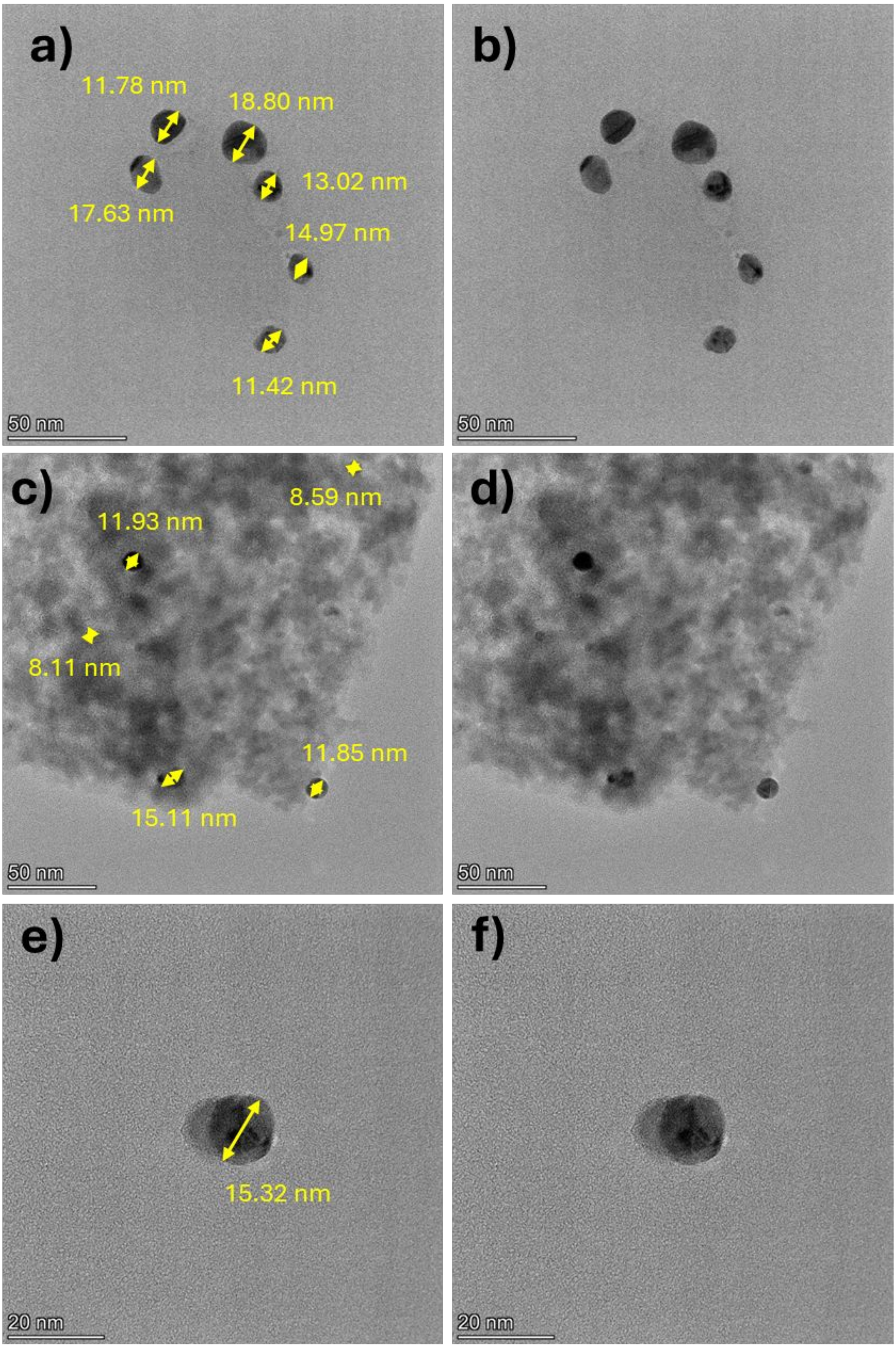

Figure S3. TEM micrographs (a, b, c, d, e, f) and particle average diameter of the Ag for Ag/SiO_2 nanofiller dispersed in isopropyl alcohol. The Ag particles diameter was measured using ImageJ software. The Ag nanoparticles can be

found isolated with sizes diameters above 12 nm, in the case of Ag mixed with SiO_2, nanoparticles with smaller diameters (8 nm) can be seen.

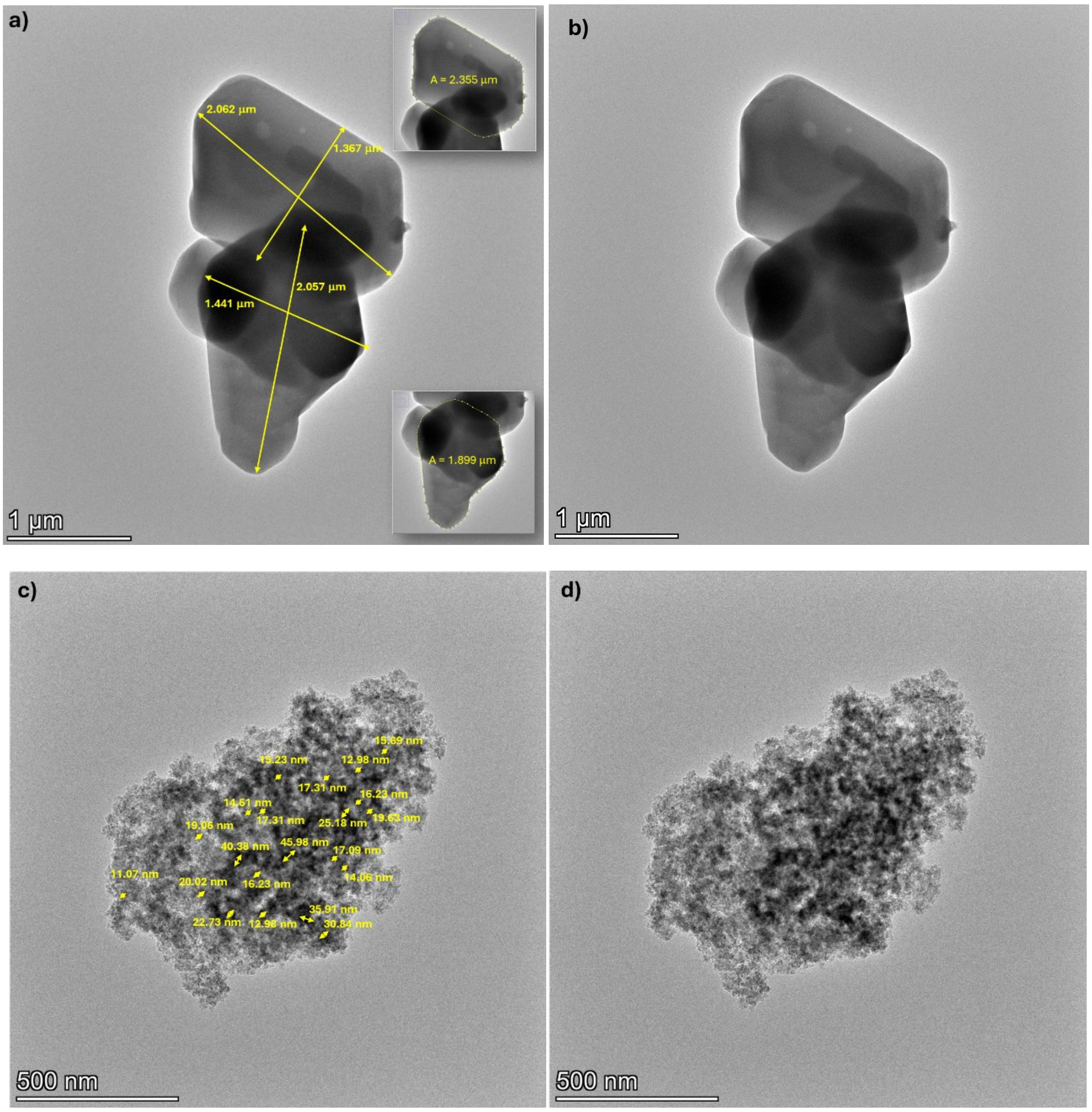

Figure S4. TEM micrographs of the (a, b) Zn particles with dimensions (area and side size) and Ag/SiO_2 for Ag/SiO_2/ZPT/TCS dispersed in isopropyl alcohol, (c, d) The Ag particles diameter was measured using ImageJ software. The Ag nanoparticles can be found mixed with SiO_2, nanoparticles show minimum diameters (10 nm) and maximum diameter 46 nm.

SEM of the PVC composites

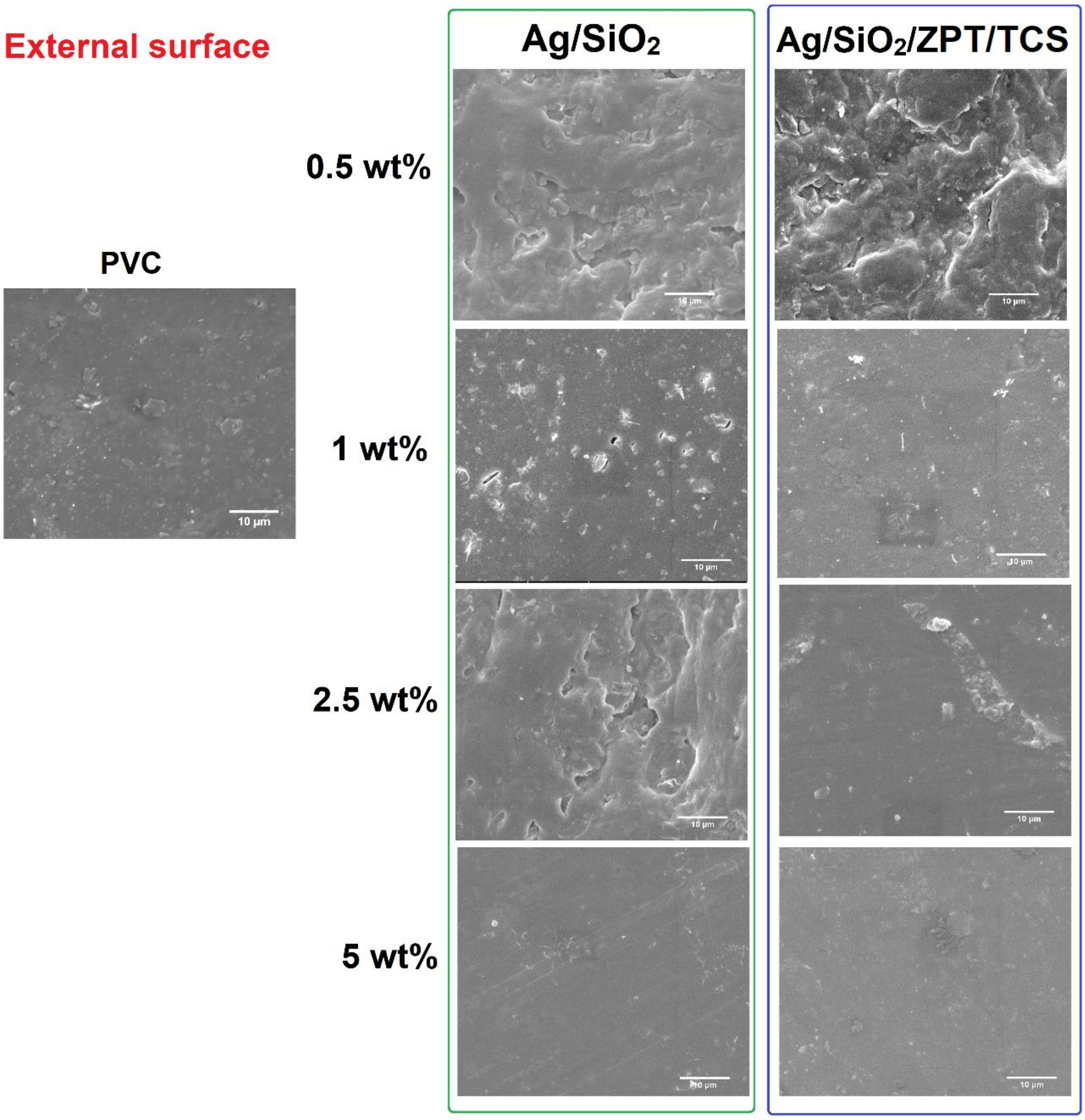

Figure S5. SEM images of the PVC, PVC/X(Ag/SiO_2), and PVC/X(Ag/SiO_2/ZPT/TCS) samples, where X corresponds to the concentration of the antiviral agent system (Ag/SiO_2 or Ag/SiO_2/ZPT/TCS). Images were obtained from the external surface of the samples.

UV-Vis diffusive reflectance spectroscopy

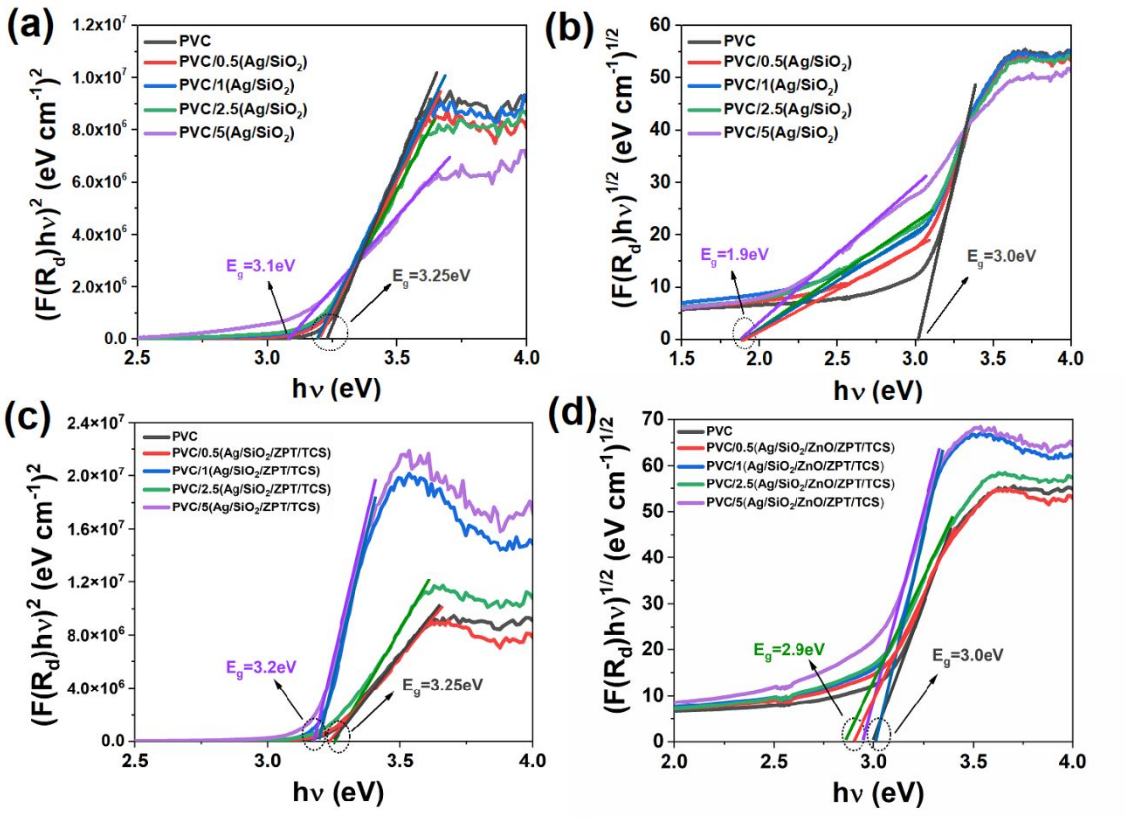

Figure S6. Tauc's plot for the direct electronic transitions of (a, b) PVC/*X*(Ag/SiO_2), and (c, d) PVC/*X*(Ag/SiO_2/ZPT/TCL) samples, where *X* corresponds to the concentration of the antiviral agent system (Ag/SiO_2 or Ag/SiO_2/ZPT/TCS).

Mechanical properties

Table S3. Tukey's test for Young's modulus (E), impact strength, and ultimate tensile strength (σ_{max}) of the PVC samples. Comparison between PVC, PVC/X(Ag/SiO_2), and PVC/X(Ag/SiO_2/ZPT/TCS) polymeric systems by ANOVA two-way.

Interaction between different polymeric systems	E (GPa)	σ_{max} (MPa)	Impact strength ($J\ m^{-1}$)
PVC x PVC/*X*(Ag/SiO_2)	≠	=	=
PVC x PVC/*X*(Ag/SiO_2/ZPT/TCS)	≠	≠	=
PVC/*X*(Ag/SiO_2) x PVC/*X*(Ag/SiO_2/ZPT/TCS)	≠	≠	≠

The signs = and ≠ mean that the properties are statistically equal and different, respectively, with a confidence level of 95 %.

ICP-MS operating parameters for Aqueous Release of Organic and Inorganic Species

Table S4. ICP-MS operating conditions for silver and zinc quantification.

**Apparatus**	**Condition/Operation**
Nebulizer	Mira Mist
Spray chamber	Scott-double pass
Torch	Quartz (1.5mm)
RF Power (W)	1550
Argon (L min-1)	Nebulizer: 1.01<br>Plasma: 15<br>Auxiliary: 0.28
Collision cell	Helium (purity>99.999%)<br>He at 5ml.min-1 or HEHe at 10ml.min-1
Interface	Platinum cones<br>Sampler: 0.9 mm<br>Skimmer: 0.45 mm
Isotopes (mode)	66Zn [He],107Ag [He]
Internal Standard	72Ge
Scan Mode	Peak hoping
Integration Time	200 (He)
Sweeps	100
Replicates	3
Reading	1
Element	Quantification Limit
Ag	0.06 ug.L
Zn	1.12 ug.L
